# Structure–Activity Study, Characterization, and Mechanism of Action of an Antimicrobial Peptoid D2 and Its d- and l-Peptide Analogues

**DOI:** 10.3390/molecules24061121

**Published:** 2019-03-21

**Authors:** Ines Greco, Johannes E. Hansen, Bimal Jana, Natalia Molchanova, Alberto Oddo, Peter W. Thulstrup, Peter Damborg, Luca Guardabassi, Paul R. Hansen

**Affiliations:** 1 Department of Drug Design and Pharmacology, Faculty of Health and Medical Sciences, University of Copenhagen, Universitetsparken 2, 2100 Copenhagen, Denmark; ines.greco@food.ku.dk (I.G.); johanneselton@hotmail.com (J.E.H.); natalia.molchanova@sund.ku.dk (N.M.); albi.oddo@gmail.com (A.O.); 2 Department of Veterinary and Animal Sciences, Faculty of Health and Medical Sciences, University of Copenhagen, Stigbøjlen 4, 1870 Frederiksberg C, Denmark; bimal@sund.ku.dk (B.J.); pedam@sund.ku.dk (P.D.); lg@sund.ku.dk (L.G.); 3 Department of Chemistry, University of Copenhagen, Universitetsparken 5, 2100 Copenhagen, Denmark; pwt@chem.ku.dk; 4 Department of Pathobiology and Population Sciences, The Royal Veterinary College, Hawkshead Lane, North Mymms, Hatfield, Herts AL9 7TA, UK

**Keywords:** antimicrobial, peptide, peptoid, mode of action, canine infections, methicillin-resistant *Staphylococcus pseudintermedius*, *Pseudomonas aeruginosa*

## Abstract

Methicillin-resistant *Staphylococcus pseudintermedius* (MRSP) constitutes an emerging health problem for companion animals in veterinary medicine. Therefore, discovery of novel antimicrobial agents for treatment of Staphylococcus-associated canine infections is urgently needed to reduce use of human antibiotics in veterinary medicine. In the present work, we characterized the antimicrobial activity of the peptoid **D2** against *S. pseudintermedius* and *Pseudomonas aeruginosa*, which is another common integumentary pathogen in dogs. Furthermore, we performed a structure–activity relationship study of **D2**, which included 19 peptide/peptoid analogs. Our best compound **D2D**, an all d-peptide analogue, showed potent minimum inhibitory concentrations (MICs) against canine *S. pseudintermedius* (2–4 µg/mL) and *P. aeruginosa* (4 µg/mL) isolates as well as other selected dog pathogens (2–16 µg/mL). Time–kill assays demonstrated that **D2D** was able to inhibit MRSP in 30 min at 1× MIC, significantly faster than **D2**. Our results suggest that at high concentrations **D2D** is rapidly lysing the bacterial membrane while **D2** is inhibiting macromolecular synthesis. We probed the mechanism of action at sub-MIC concentrations of **D2**, **D2D**, the l-peptide analog and its retro analog by a macromolecular biosynthesis assay and fluorescence spectroscopy. Our data suggest that at sub-MIC concentrations **D2D** is membrane inactive and primarily works by cell wall inhibition, while the other compounds mainly act on the bacterial membrane.

## 1. Introduction

Canine skin infections and otitis constitute the number one reason for antimicrobial prescription in dogs. *Staphylococcus pseudintermedius* is the main canine pathogen associated with these conditions [1]. In recent years, the spread of methicillin-resistant *Staphylococcus pseudintermedius* (MRSP), such as the epidemic clone sequence type ST68 in North America and ST71 in Europe, has become a serious therapeutic challenge in small animal medicine [2,3]. Also, human infections associated with MRSP ST71 have been reported [4]. Although less frequent than *S. pseudintermedius*, *Pseudomonas aeruginosa* is another pathogen associated with canine pyoderma as well as infections of the canine ear canal [5]. This pathogen may also constitute a treatment challenge due to its intrinsic resistance to several antimicrobial agents. The shortage of antimicrobial options against resistant integumentary infections stresses the need for new antimicrobials specifically for veterinary medicine [6]. 

Antimicrobial peptides (AMPs), also known as host defense peptides, are present in a broad variety of multicellular organisms [7]. As they contain both hydrophobic and positively charged residues, AMPs amphipatic nature provides a preferential cell selectivity towards bacterial cells rather than eukaryotic cells [8]. To date, AMPs are mostly believed to act through a fast, membrane-disrupting bactericidal mechanism [9]. Whereas peptides have an undeniable potential to become a base for the next generation of antimicrobial agents, they exhibit a number of limitations, such as low bioavailability due to their susceptibility to proteases [10]. In order to overcome such stability issues, several classes of synthetic peptides, or peptidomimetics, which mimic the structure and function of AMPs, have been introduced in the past decades [11]. These classes of molecules retain the antimicrobial activity and mechanism of action of AMPs, while gaining stability to proteases. Peptoids, constitute a class of the peptidomimetics that exhibit promising antimicrobial activity and protease stability [12,13]. Notably, when introduced into the peptide, peptoid residues can improve the antibacterial profile of the parent peptide alongside its biostability, yielding promising peptide–peptoid hybrids [14,15,16]. Though AMPs and their mimics often display high systemic toxicity issues [17], they, alone or in combination with a conventional antibiotic have potential to become therapeutics for topical application, e.g., to treat bacterial skin infections. 

We have previously described a promising antimicrobial peptoid, **D2**, which is active against MRSP and *P. aeruginosa*, resistant to plasma proteases and hepatic clearance in vitro, and is suitable for topical administration to treat superficial pyoderma [18]. In the present study, we investigated the structural features responsible for antimicrobial activity of **D2** by (a) performing Gly-scan to analyze the residues crucial for activity; (b) investigating the effects of single substitution in order to increase activity; (c) exploring the effect of the peptoid backbone by exchanging l- and d- amino acids for their peptoid counterparts; (d) testing against a panel of canine pathogens; (e) performing circular dichroism studies; (f) measuring the biosynthesis rate of macromolecules and bacterial proton motive force to elucidate the mode of action.

## 2. Results and Discussion

### 2.1. MIC Distribution of **D2** and Time–Kill Kinetics

**Figure 1:**
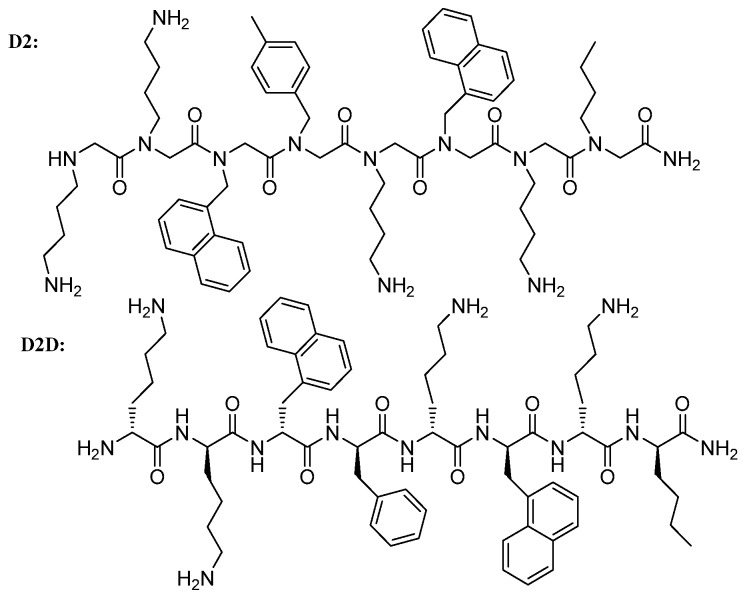
Lead compound **D2** and its most active analogue **D2D**.

The potential of **D2** (Figure 1) to treat infections topically has been studied previously, including release from formulation and in vitro ADME (absorption, distribution, metabolism, and excretion) properties [18]. In the present study, we further evaluated the antimicrobial activity of **D2** against a panel of 50 *S. pseudintermedius* and 50 *P. aeruginosa* isolates from canine infections (Figure S2 Supporting information). The MICs ranged from 1.56 to 3.12 µM and 3.12 to 12.5 µM, respectively.

**Figure 2:**
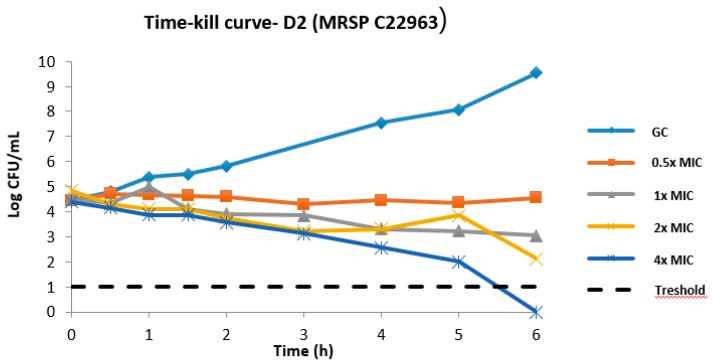
Time–kill curve of **D2** against a clinical MRSP isolate (C22963). GC: Growth control. We observed regrowth after 24 h (data not shown).

D2 time–kill experiments were done using the clinical MRSP strain C22963 (Figure 2). At 1× and 2× MIC, we observed log 1.5 and log 2.7 reductions after 6 h.

The MICs of **D2** are comparable with previous literature reports on peptides targeting MRSP. Molchanova et al. described a number of different α-peptide/β-peptoid hybrids, which were active against MRSP (2–8 µg/mL) as well as other pathogenic bacteria relevant to human and veterinary medicine [15]. In continuation of the above study, the same authors investigated the effect of fluorination, oligomer length, and end-group modification [19]. These peptidomimetics were tested against *S. pseudintermedius* isolates, with MICs ranging from 0.5 µg/mL to 4 µg/mL. The compounds were also potent against *Escherichia coli*, *P. aeruginosa*, and Methicillin-resistant *Staphylococcus aureus* (MRSA). The antimicrobial activity of six peptides against clinical isolates of Methicillin-susceptible *Staphylococcus pseudintermedius* (MSSP) and MRSP from infected dogs was also investigated by Mohamed et al. [20]. The most potent compounds had a MIC_50_ and MIC_90_ of 1 and 2 µM, respectively. Very recently, we reported a peptide–peptoid hybrid, **B1**, which showed potent MICs against a number of canine *S. pseudintermedius* (2–4 µg/mL) and *P. aeruginosa* (8–16 µg/mL) isolates [21]. This compound inhibited MRSP (C22963) and *P. aeruginosa* in less than 30 min at 8× MIC as shown by time–kill kinetics. The peptoid **D2** showed slower killing kinetics compared to **B1** and antimicrobial peptide and peptoid analogues of **B1**. Finally, another peptide, AMP2041, showed LD_90_ values of 0.5–8 µg/mL against *P. aeruginosa* strains derived from dog otitis [22].

### 2.2. Structure–Activity Relationship (SAR) Study of **D2**

In order to understand the structural features of **D2** responsible for its activity, we have performed a structure–activity relationship study on its backbone. First, we performed a Gly-scan by substituting sequentially every peptoid residue with a Gly amino acid, to elucidate the effect of every side chain on the activity of **D2**. We identified the side chain in position 5 as not essential for antimicrobial activity and proceeded substituting this residue with other residues.

#### 2.2.1. Glycine-Scan of **D2**

Glycine-scan technique corresponds to the systematic replacement of each residue by glycine. Such structure–activity relationship study provides information on the functional role of the individual residues in the peptoid. This includes the contribution of the single peptoid residue side chain to the biological activity of **D2**. Furthermore, hydrophobic and electrostatic side-chain interactions as well as H-bonds with the target may be probed [23]. A total of 8 glycine analogs of **D2** (**1**–**8**) were synthesized with systematic substitutions of glycine in every position (Table 1).

The glycine scan revealed that the bulky hydrophobic aromatic groups such as 1-naphthalenemethyl in position 3 and 6, and 4-methylbenzyl in position 4 were crucial for antimicrobial activity, as their removal was accompanied by a shift in MIC values to 64 μg/mL or higher against all tested strains. Notably, Gly to Nleu substitution in position 8 had a less detrimental impact on antimicrobial activity. The loss of antimicrobial activity for these analogs is not surprising, considering that hydrophobicity is one of the crucial parameters for antimicrobial activity of AMPs and peptidomimetics. The ability of AMPs to lyse red blood cells, also referred to as hemolysis, is often used as an indication of toxicity [24]. Decrease in the hydrophobicity is often associated with a simultaneous decrease in hemolytic activity; however, for the NMePhe to Gly and Nle to Gly substitutions in positions 4 and 8, respectively, the opposite effect was observed, with an approximately two-fold increase in HA%. Surprisingly, the substitutions of N1Nal in positions 3 and 6 led to opposite effects on the HA% (56% and 6% respectively).

The NLys substitution in position 2 resulted in a 2–4-fold loss of activity against all strains tested, and a 2-fold increase in HA%. This suggests a high importance of a cationic residue in this position. The NLys substitutions in position 1, 5, and 7 had a subtler impact on antimicrobial activity: no activity loss was observed against MSSP and MRSP, but a slight decrease of activity against *P. aeruginosa* and *S. aureus* was observed for all three analogs. NLys substitution in the position 1 resulted in a 2-fold increase in hemolysis at 150 μM, while no effect was observed for the NLys in position 5. Interestingly, the NLys substitution in position 7 increased the HA at 150 μM dramatically to 85%.

#### 2.2.2. Substitutions in Position 5

The glycine scan revealed that the *N*Lys residue in position 5 was non-essential for both antimicrobial and hemolytic activity, suggesting it to be a potential site for optimization. Hence, a series of peptoid analogues of **D2** were designed, where *N*Lys residue was substituted with four different types of side chains (**9**–**12**) in position 5. Four side chains were chosen to represent a wide spectrum of hydrophobicity, from short aliphatic side chains such as methyl to bulkier aromatic groups such as 4-methylbenzyl and 1-napthalenmethyl. An analog with a polar NEtOH group as side-chain was also included (**12**), as seen in Table 2.

For all tested compounds, little to no effect in antimicrobial activity was observed compared to the lead peptoid **D2**. The analogue with methyl side chain (**9**) maintained MIC values of 4 µg/mL against both MSSP and MRSP. Analogues **10** and **11** with bulky side chains showed a slight increase in MIC from 4 to 8 µg/mL. No significant change in selectivity was observed against *S. aureus* or *P. aeruginosa*.

We found that the side chain modifications in position 5 had a more profound impact on the hemolytic activity. A clear correlation between increasing hydrophobicity and hemolytic activity was observed for all analogs, with peptoid **11** being the most hemolytic compound. Interestingly, a short polar aliphatic side chain in position 5 of analogue **12** caused a reduction in the hemolytic activity to 5%, however at a slight cost of antimicrobial activity. Since little or no change in antimicrobial activity was observed for all analogs, we speculate that the hydrophobicity threshold for **D2** was reached.

### 2.3. Modification of Backbone Nature of **D2**

The presence of peptoid residues in the backbone of **D2** could be another contributing factor for its antimicrobial and hemolytic activity. To investigate the extent of this effect, we generated the corresponding l- and d- peptide analogues of **D2** (**D2L** and **D2D**). Furthermore, we generated an l-retro-peptide analogue (with inverted sequence) and a series of peptide–peptoid hybrids by systematically replacing charged or hydrophobic peptoid residues with corresponding d-residues.

All three peptide analogs of peptoid **D2** were designed either using only l-amino acids (**D2L**) or d-amino acids (**D2D**), and added a reversed sequence using only l-amino acids (**D2R**) (Table 3).

Both l-peptides **D2L** and **D2R** displayed a general loss of activity against all bacterial strains, most notably against MSSP (with a 4–8-fold decrease in antimicrobial activity). Interestingly, the analog **D2D** containing only d-amino acids demonstrated a 2–4-fold improvement in activity against both S. aureus and P. aeruginosa, while maintaining activity against MSSP and MRSP. Peptides **D2L** and **D2D** exhibited similar hemolytical activities (35% at 150 µM). Retro-peptide **D2R** showed hemolytic activity (23% at 150 µM) comparable to that of the lead peptoid **D2** (24%). Although the initial aim of the project was to optimize **D2** against MRSP, the increased broad-spectrum activity observed for analog **D2D** made this peptide an interesting lead compound.

### 2.4. C- and N-Substituted Analogs

Many studies have found that interruption of the hydrophobic surface and/or disruption of amphipathic secondary structure primarily impacts the toxicity of AMPs to eukaryotic cells, while having more moderate effects on their antimicrobial activity [25]. As the next step, in the effort to reduce the hemolytic activity of the analog **D2D**, four peptide/peptoid hybrids were designed by systematically replacing peptoid residues with the corresponding d-amino acids. In analogs **13** and **14**, C-terminal and N-terminal residues of peptoid **D2** were replaced with d-amino acids, respectively. In analogs **15** and **16**, the hydrophobic or hydrophilic residues were replaced with the corresponding d-amino acids, respectively (Table 3).

Introduction of d-amino acids in the sequence resulted in maintaining activity against Gram-positive bacteria. Accordingly, the MIC values against *S. pseudintermedius* remained unaltered with all the substitution tested. The substitution of d-amino acids with peptoids in the full N-terminal moiety of **14** did not show any effect on the activity on *S. aureus*, while the C-terminal substituted **15** and the hybrids **16** lost activity against *S. aureus* (MICs of 16 to 32 µg/mL). Three peptoid–peptide hybrids (**14**, **15**, and **16**) lost activity against *P. aeruginosa* with MICs increasing from 16 µg/mL to 64 µg/mL or more. With regard to hemolysis, while analogs **14**, **15**, and **16** showed no detectable hemolytic activity at 150 µM, hybrid **13** demonstrated hemolysis comparable to the analog **D2D** (34%), the all d-amino acid peptide version of **D2**.

### 2.5. Antimicrobial Activity of the Best Analogue **D2D**

#### 2.5.1. Time–Kill Kinetics of **D2D**

**Figure 3:**
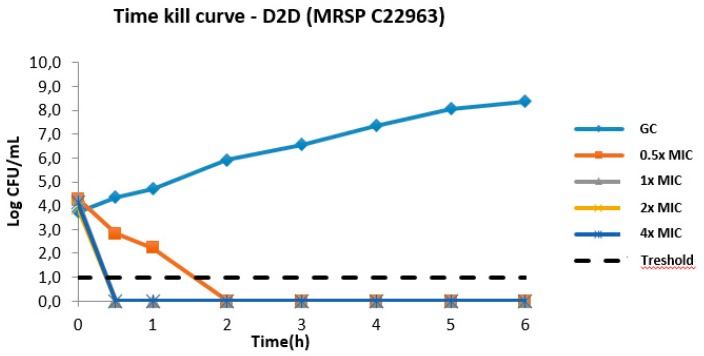
Time–kill curves of **D2D**, the best candidate analogue identified in this study, against a clinical MRSP isolate (C22963). GC: growth control. We observed regrowth after 24 h (data not shown).

In the light of the results above, **D2D** was chosen for time kill kinetic studies against a clinical MRSP strain (C22963, Figure 3). The all-d peptide showed killing to bacterial levels below the detection threshold at 1× MIC in 30 min. Furthermore, a clear concentration-dependent bacterial killing was observed, as are often seen for cationic AMPs [11].

#### 2.5.2. Antimicrobial Activity of **D2** and **D2D** against Canine Pathogens

We screened the antimicrobial activity of **D2** and the analogue **D2D** against a number of canine pathogens (Table 4). These included *Acinetobacter baumannii*, *Escherichia coli*, *Enterococcus faecalis*, and *Klebsiella pneumoniae* isolated from wounds, *Corynebacterium auriscanis* associated with canine otitis externa [26], the nosocomial pathogen *Enterococcus faecium* [27], the endocartitis-causing *Streptococcus canis* [28], and *Proteus mirabilis* [29]

**D2** only showed activity against *C. auriscanis* (MIC = 2 µg/mL) and *E. faecium* (MIC = 16 µg/mL). MICs for **D2D** were ranging from 2–16 µg/mL, except for *P. mirabilis* (64 µg/mL). In the majority of cases, **D2D** showed a 2- to 8-fold better activity than **D2**. Our MIC data for **D2D** are fully comparable with previous literature reports [21].

#### 2.5.3. MSSP vs. MSSA

Very recently, we reported two structurally related peptides, which showed preferential activity against *S. pseudintermedius* (MIC = 2–16 µg/mL) over *S. aureus* (MIC = 32–64 µg/mL) [21]. Similarly, in this study, to assess the degree of selectivity for *S. pseudintermedius,* **D2** and **D2D** were tested against a collection of *S. aureus* and MSSP strains (Table S2). However, **D2** and **D2D** retained comparable activity against multiple clinical strains of MSSA (1–2 µg/mL) and MSSP (2–8 µg/mL).

### 2.6. Circular Dichroism of Selected Analogues of **D2**

In order to investigate the solution folding of the **D2** peptide analogues in Table 3, circular dichroism (CD) spectroscopy was applied. The compounds **D2L**, **D2D**, **D2R**, and the analogs **13**–**16** were studied in 10 mM phosphate buffer at pH 7.4 with and without addition of 50% (*v*/*v*) TFE (2,2,2-trifluooethanol) (Figure S2). The far UV CD spectra were expected to have a strong influence from the aromatic groups, and the spectra of **D2L** do bear a resemblance to the far UV signals observed for *N*-acetyl-l-1-naphtylalanine ester [30], which has a positive component at 240 nm, a negative at 220 nm and again a positively signed signal below 200 nm. Thus, the all l- peptide **D2L** showed a positive band above 225 nm, two negatively signed bands at 208 and 215 nm, and changes to a positive signal below 200 nm. Interestingly, the co-solvent TFE—a simple membrane model, which is known to induce α-helical folding—changed the peptide conformation and yielded much stronger CD signals (Figure S4). If **D2R**—the retro sequence with l-amino acids—is compared to **D2L**, it appears that peptide backbone conformation does play a role for the CD spectra. We thus interpreted the spectral changes in TFE to correspond to an increase in folded conformers, likely with α-helical content. Spectral overlap with aromatic side-chain transitions may via sign-cancellation partially obscured the amide *n*-π* band at 222 nm that is characteristic of α-helical folding. As expected, the all d-enantiomer **D2D**, showed a mirrored spectral signature to the **D2L**. The presence of the peptoid residues disrupted the peptide structure as hybrid analogs **13**, **14**, **15**, and **16** all showed spectra with limited secondary-structure signatures, and with no significant differences appearing by changing solvent from aqueous buffer to 50% TFE (Figure S4).

### 2.7. Investigating the Mechanism of Action by Emission DiSC3(5) Fluorescence and Macromolecule Biosynthesis Assay

Cationic antimicrobial peptides act cooperatively, accumulating on the negatively charged membrane. When a threshold concentration is reached, AMPs disrupt the membrane, causing membrane thinning and creating transient pores at intermediate concentrations [31]. However, AMPs can translocate into bacterial cells through the transient pores [32] at sub-MIC concentrations without causing lethal membrane disruptive events and interacting with intracellular targets [33].

In this study, we probed the mechanism of action of **D2** and selected analogues using fluorescence spectroscopy and macromolecule biosynthesis assay at sub-inhibitory concentrations. Most of the peptides may induce cell lysis at higher concentration but our aim was to identify the novel mode of action if that exist beyond cell lysis, and for that use of sub-MIC concentration is very important The relative change of membrane proton motive force (PMF) is often measured by fluorescence spectroscopy using a fluorophor, which is concentrated in the bacterial membrane [34]. If the AMP is membrane active at sub-inhibitory concentrations, the probe is released into the assay medium. AMP inhibition of DNA and cell wall synthesis are probed by measuring the rate of incoorporation of the radiolabeled precursors ^3^H-thymidine and ^3^H-Glucosamine hydrochloride, respectively [35].

#### 2.7.1. Growth Curve Analysis

First, we performed growth curve analysis using the well-characterized MRSP ST71 strain E104, which is resistant to a number of antibiotics [36]. Growth curve experiments showed decrease of cell optical density (OD) over time indicating cell lysis by **D2**, **D2L**, **D2D**, and **D2R** at the MIC (Figure S3 Supporting Information). Only minor cell growth inhibition was observed at sub-MIC concentrations. A well-characterized antimicrobial peptide, nisin, was used as control. Nisin interacts with the lipid II layer and consequently inhibits cell-wall synthesis as shown in a number of biophysical studies, including NMR [37], isothermal titration calometry [38], and dye leakage experiments [39]. Increased concentrations of nisin inhibited proportionally the growth of E104, both at sub-MIC and MIC concentrations.

#### 2.7.2. Emission DiSC3(5) Fluorescence Spectroscopy

**Figure 4:**
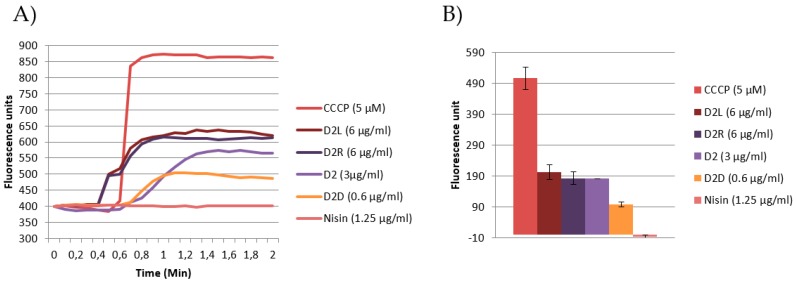
Effect on membrane depolarization at sub-MIC concentrations (0.5× MIC) of the positive control CCCP, **D2**, **D2D**, **D2L**, **D2R**, and the negative control nisin. (**A**) Intensity of fluorescence (arbitrary units) is reported as function of the time; (**B**) change of fluorescence after 1.6 min for each compound ± SEM (n = 2).

In this study, the DiSC3(5) probe was used to assess the effect of **D2** and its structural analogues **D2L**, **D2D**, and **D2R** on cytoplasmic membrane depolarization. DiSC3(5) is a cationic carbocyanine dye, which accumulates on negatively charged membrane, translocates into lipid membrane with a distribution dependent on plasma and mitochondrial membrane potential [40]. Altered emission profile of DiSC3(5) measured via fluorescence spectroscopy indicates membrane depolarization caused by proton motive force dissipation, ATP production and/or pore formation. The increment of DiSC3(5) fluorescence of labeled cells resulting from its leakage upon addition of antimicrobial agents is usually measured over time [40]. The protonophore carbonyl cyanide metachlorophenyl hydrazone (CCCP) is used as positive control, which rapidly dissipates PMF increasing proton permeability, alters both membrane proton gradient and transmembrane electric potential and decreases cellular ATP content [41]. Based on the MIC and growth analysis results, sub-lethal concentrations of **D2** and selected analogues were chosen to perform DiSC3(5) fluorescence and macromolecule biosynthesis studies. **D2L** and **D2R** showed the strongest effect on PMF followed by the peptoid **D2** and **D2D** (Figure 4). The peptoid **D2** is nearly as active as **D2L** and **D2R**, suggesting that cationicitiy and hydrophobicity is important for membrane interaction. Nisin does not have any effect on PMF.

#### 2.7.3. Macromolecule Biosynthesis Assay

The effect on DNA and cell wall synthesis was investigated after 20 min of incubation of MRSP E104 with the precursors ^3^H-thymidine and ^3^H-glucosamine hydrochloride, respectively, as previously reported by Ling et al. [42]. Following macromolecule precipitation, radioactivity was counted using scintillation fluid. Incorporation of ^3^H- or ^14^C-labeled thymidine has been used to investigate DNA replication and transcriptional activity during the cell cycle [43]; ^3^H-glucosamine hydrochloride has been used in a mode of action study for the antimicrobial peptide plectasin, which targets the cell wall precursor lipid II [44].

**Figure 5:**
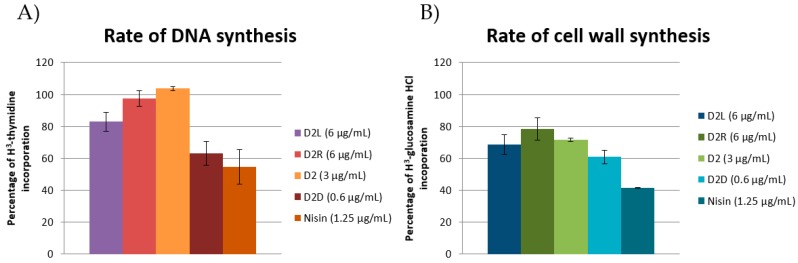
Effect of sub-MIC concentration of AMPs on macromolecule biosynthesis. (**A**) Effect on DNA and (**B**) cell wall synthesis in cells treated with sub-MIC concentrations (0.5× MIC) of **D2D**, **D2L**, and **D2R** after 20 min of incubation. The peptide nisin was used as control. Data are represented as means of two repeated experiments ± SEM.

AMPs have been reported to interact with DNA [45], being both cationic and hydrophobic. The most active compound in the DNA biosynthesis rate assay (Figure 5a) was **D2D** (37% inhibition) followed by **D2L** (17% reduction in DNA replication). The compounds **D2** and **D2R** did not show any significant activity (<5%). For nisin, a 45% reduction was observed. Our finding that nisin shows no membrane depolarisation but is the most significant at inhibiting macromolecular synthesis is in contrast with previous reports. At the concentrations used in this study, nisin is known to bind to lipid II and forms pores [46] effectively operating through a receptor-mediated membrane lysis mechanism. **D2D** demonstrated the most pronounced effect on cell wall synthesis (39%, Figure 5b) followed by **D2L** (31%), **D2** (28%), and **D2R** (22%).

The data reported in this study suggest that **D2D** has an intracellular mode of action. Not only is **D2D** the compound with the lowest activity on PMF among all the tested compounds (Figure 4), but at sub-MIC concentration it also inhibits DNA and cell wall synthesis (Figure 5a,b). This is in agreement with previous reports that some AMPs may inhibit DNA and cell wall synthesis, including indolicidin [45].

Our finding that the d-peptide is more active that the l-enantiomer is not surprising. The difference underlying this reason is unclear; however it could be attributed to a higher resistance of the d- analogue to bacterial proteases or to increased affinity towards bacterial membrane targets. Since **D2D** is approximately four-fold more active than its enantiomer **D2L**, we cannot rule out that **D2D** is binding to a chiral receptor and then causing membrane lysis/pore formation. In a recent study, the activity and mode of action of a sapesin B analog, KLKLLLLLKLK-NH_2_, have been compared to its corresponding all-d analogue [47]. The d-peptide showed a significantly higher antimicrobial activity against *S. aureus* (1 vs. 16 µg/mL). The authors found that this is due to its increased affinity to peptidoglycan, and suggested that the peptide chiral components of the peptidoglycan could be involved in this preferential interaction. Similar results were reported by Oddo et al. [48]. It is likely that the fast kill kinetics showed by **D2D** could be attributed to a combined effect on the membrane and of interaction with DNA and cell wall synthesis, which might be related to a higher affinity of **D2D** for macromolecules.

We observed that the retro-l-peptide has approximately the same MIC and membrane activity as the l-peptide. However, the l-analogue inhibits DNA and cell wall synthesis to a higher degree, indicating the distribution of residues in the chain may play a significant role in the activity.

The membrane activity of the full peptoid **D2** appears to be between the d- and l-peptide form. The peptoid did not show any effect on DNA synthesis and its effect on cell wall synthesis was comparable to the peptide **D2L**. This suggests that backbone hydrogen bonding is important in DNA synthesis inhibition. Furthermore, the different conformational flexibility of peptoids compared to peptides may influence their interaction with the bacterial membrane. For example, it has been reported that flexible non-natural antimicrobial peptidomimetics act differently on the bacterial membrane compared to their structured analogues [49].

The results obtained in this study about **D2** are in agreement with a peptide–peptoid hybrid, **B1**, we reported recently [21]. This peptidomimetic also did not inhibit DNA synthesis and showed cell wall synthesis inhibition.

Insertion of even a single peptoid residue in an antimicrobial peptoid may change the activity and mode of action. For example, using membrane depolarization, dye leakage experiments and confocal data, Jeong et al. demonstrated that peptoid-containing analogues of Piscidin-1 (FFHHIFRGIVHVGKTIHRLVTG) penetrated the cell membrane of *S. aureus*, indicating the presence of intracellular targets. In contrast, the corresponding peptides permeabilized specifically the bacterial cell membrane [50]. Finally, two short linear peptidomimetics with a modular structure of peptoid residues resembling tryptophan and lysine have been recently reported by Mojsoska et al. [51]. Both peptoids caused membrane permeabilization in *E. coli*, to different degrees between the two molecules.

## 3. Materials and Methods

### 3.1. Materials

Disposable 5-mL polypropylene reactors fitted with a PTFE filter were acquired from Thermo Fisher Scientific (Hvidovre, Denmark). Tentagel S RAM resin, TFA, piperidine, and Fmoc-protected l- and d-amino acids were purchased from Iris-Biotech GmbH (Marktredwitz, Germany) Primary amines, bromoacetic acid, DIEA and Triisopropylamine were from Sigma-Aldrich (Søborg, Denmark). HOAt (1-Hydroxy-7-azabenzotriazole) and HATU (1-[Bis(dimethylamino)methylene]-1*H*-1,2,3-triazolo [4,5-b]pyridinium 3-oxid hexafluorophosphate, *N*-[(Dimethylamino)-1H-1,2,3-triazolo-[4,5-b]pyridin-1-ylmethylene]-*N*-methylmethanaminium hexa-fluorophosphate N-oxide) were from GL Biochem (Shanghai, China). DMF (dimethylformamide, synthesis grade), DCM (dichloromethane, optical grade), MeCN (acetonitrile, optical grade) were from VWR (Copenhagen, Denmark). All reagents and solvents were used without further purification.

### 3.2. Synthesis

The peptides **D2D**, **D2L**, and **D2R** were synthesized manually on a Tentagel S RAM resin (0.22 meq/g) in a syringe equipped with a fritted filter. Fmoc amino acids were coupled using HATU, HOAt and DIEA (4:4:8 eq) in DMF for 2 h. Deprotection was accomplished with 20% DMF in piperidine (3 × 4 min). The peptoid **D2** was synthesized on a TentaGel S RAM resin using the submonomer approach. Peptoid residues were coupled using bromoacetic acid and DIC (1:1, 10 equiv.) in DMF for 30 min followed by amine (40 equiv.) displacement in DMF for 2 h. Peptoid hybrid synthesis was done using a combination of the submonomer approach and Fmoc SPPS described above. Following synthesis, the product was cleaved from the resin with TFA:H_2_O:TIS (95:2.5:2.5), precipitated in ether and lyophilized. Peptide purification was achieved by preparative reverse-phase HPLC system consisting of Waters^TM^ (Milford, MA, USA) 600 Pump, In-line Degasser, 600 Controller and 2996 Photodiode Array Detector, the column used was a Waters^TM^ XSelect^®^ Peptide CSH C18 OBDTM, 5 µm, 19 × 250 mm with H_2_O:ACN gradient. The appropriate fractions were concentrated and lyophilized. Purity was determined by analytical reverse-phase HPLC system consisting of Waters^TM^ 717 plus Autosampler, in-line degasser AF, 600 controller and 2996 photodiode array detector, the column used was a Waters^TM^ Symmetry^TM^ C18, 5 µm, 4.6 × 250 mm on an acetonitrile-water gradient. Finally, the products were characterized by matrix-assisted laser desorption/ionization time-of-flight mass spectrometry (Bruker Microflex, Bremen, Germany), using α-cyano-4-hydroxycinnamic acid as matrix.

### 3.3. Antimicrobial Susceptibility Testing

To explore the antimicrobial activity of the library of peptides and peptidomimetics, they were tested against *Acinetobacter baumannii*, *Corynebacterium auriscanis*, *Escherichia coli*, *Enterococcus faecalis*, *Enterococcus faecium*, *Klebsiella pneumonia*, *Proteus mirabilis*, and *Pseudomonas aeruginosa*. Bacteria grown on agar plates for 18 h at 37 °C were diluted to ~1 × 10^8^ CFU/mL in Mueller–Hinton broth (MHB). Two-fold serial dilutions of peptides and peptidomimetics in MHB II were inoculated with bacteria to achieve a final concentration of 5 × 10^5^ CFU/mL in polypropylene 96 U-well microtiter plates (Almeco, Esbjerg, Denmark), followed by incubation at 37 °C in ambient air for 18 h. The MIC values were determined as the lowest concentration showing no visible bacterial growth. Experiments were performed twice (in technical triplicates) on two different days.

### 3.4. Time–Kill Kinetics

The time–kill experiments of **D2** against the methicillin-resistant *S. pseudintermedius* strain C22963 were performed using an in-house protocol adopted and modified from Blondeau et al. [52]: the bacterium was grown overnight on a blood agar plate. The next day, an inoculum was transferred to MHB and incubated for 2 h at 37 °C to reach exponential growth phase. The inoculum was then adjusted to achieve cell density of 10^5^ CFU/mL in MHB using a nephelometer, and the antimicrobial agent was added in culture tubes in 2-fold concentrations from 0.5 to 4 times the MIC. Aliquots were removed at 1 h intervals up to 6h and spotted in triplicate on blood agar plates, and incubated overnight at 37 °C. Colonies were counted and CFU per mL was calculated.

### 3.5. Hemolysis of Red Blood Cells

Hemolysis was done as described by Oddo et al. [48]. Briefly, 75 µL of peptide in PBS was mixed with 75 µL of a 0.5% red blood cell (RBC) suspension in PBS, and incubated for 1 h at 37 °C. The hemoglobin release was then measured at 414 nm and normalized, using mellitin as positive control and PBS as negative control. Experiments were done in triplicates.

### 3.6. Growth Curves

#### Performance of Growth Curves in High-Throughput

The effect of **D2** or analogues or nisin on the growth of MRSP strain E104 was evaluated using BioScreen (Oy Growth Curves Ab LTd, Helsinki, Finland). Freshly grown culture of E104 was diluted to OD 0.2 at 600 nm, supplemented with increased concentration of antimicrobials and distributed in a 100 well honeycomb micro-plate with volume 100 μL. The plate was incubated for 6 h at 37 °C with continuous shaking. The OD_600_ of each micro-culture was measured and recorded every 15 min after a 5 s pause of shaking in automatic mode. Recorded OD was plotted against time to prepare the growth curves. Experiment was performed twice and one representative result was presented.

### 3.7. Circular Dichroism (CD) Spectroscopy

CD spectroscopic experiments were used to study the solution structure of analogues in phosphate buffer (10 mM, pH 7.4) and in 50% 2,2,2-trifluoroethanol (TFE) using a Jasco J-815 spectropolarimeter in Hellma QS cuvettes with pathlength 1.00 mm. Stock solutions of 1 mg/mL were prepared from lyophilized analogs in 50 mM Na_2_HPO_4_, pH 7.4 and were diluted to appropriate concentrations of ~0.1 mg/mL in 10 mM buffer, or same concentration in 50% TFE (*v*/*v*). The spectra of the analogues were recorded in 10 mM buffer and 50% (*v*/*v*) TFE at room temperature. The measurements were conducted in the far UV range from 250 to 190 nm with a scan rate of 20 nm/min at 1 nm bandwidth, and a time constant of 0.5 s. The resulting spectra were the average of six separate recordings. Blank samples of 10 mM buffer, respectively 10 mM buffer with 50% (*v*/*v*) TFE, were recorded and subtracted from the relevant sample spectra. The CD spectra were normalized with regard to the UV absorbance at 280 nm (relative to the **D2D** analog). The UV absorbance data were measured on a Shimadzu UV-3600 UV–vis–NIR spectrophotometer using a 10 mm Hellma quartz cuvette. Finally, the CD spectra were smoothed with a five-point Savitzky–Golay filter in Jasco spectra analysis and the CD signal at 250 nm was adjusted to zero.

### 3.8. Fluorescence Assay

#### Studying Bacterial Membrane Potential by Measuring DiSC3(5) Fluorescence

Freshly grown E104 cells with OD 0.2 at 600 nm were labeled with 1 µM 3,3-dipropylthiadicarbocyanine iodide [DiSC3(5)] (Sigma-Aldrich, Søborg, Denmark) in MHB. The fluorescence spectra of fluorophore-labeled cells were plotted using the LS-50B luminescence spectrometer (PerkinElmer, Waltham, MA, USA) at excitation/emission wavelengths 546 nm/573 nm using time drive application of FLWINLAB software. After reading initial stable emission spectra of DiSC3(5), labeled cells were exposed with D2 or analogues or nisin or protonophore CCCP by directly injecting the concentrated solution of agent to the cuvette, and the change of fluorescence over time was recorded until emission spectra reached to stability. The increment of DiSC3(5) fluorescence upon addition of antimicrobials or CCCP (FUafter-exposure – FUbefore-exposure) was calculated and plotted in addition to raw fluorescence spectra. Experiment was performed twice and average fluorescence change was plotted with standard deviation.

### 3.9. Macromolecule Biosynthesis Assay

#### Assay to Measure the Macromolecule Biosynthesis Rate

Macromolecules (DNA and cell wall) biosynthesis rates were measured using strain MRSP E104 following a protocol adapted from Ling et al. [42]. Briefly, overnight culture of E104 was diluted 1:100 in MHB and freshly grown up to OD 0.2 at 600 nm. Grown cells were pelleted down by centrifugation and then resuspended in fresh MHB medium followed by incubation for 20 min on a dry heat-bath with **D2** or analogues or nisin and radiolabeled precursor: (50 µCi) ^3^H-Thymidine (PerkinElmer) and (5 µCi) ^3^H-glucosamine hydrochloride per mL for DNA and cell wall, respectively. A positive control without antimicrobials was maintained that corresponds to 100% synthesis rate. After 20 min of incubation, samples were precipitated with equal volume of cold 30% trichloroactic acid (TCA Sigma, Søborg, Denmark) on ice. After 15 to 30 min of incubation on ice, precipitates were filtered on a membrane filter and subsequently washed twice with cold 15% TCA and twice with cold water using a vacuum manifold. Next, filters were air dried overnight and then moved to 10 mL scintillation vials. Finally, 3 mL scintillation fluid was added to each vial and ^3^H count was taken in Beckman Coulter LS6500 liquid scintillation counter for 1 min. The radioactive counts of the control samples with no antimicrobial exposure were considered to have 100% precursor incorporation and macromolecule synthesis. The percentage rates of macromolecule synthesis in the antimicrobial-exposed samples were calculated compared to control. Each experiment was performed twice with a technical replicate, and the average rates of synthesis were plotted.

## 4. Conclusions

In this study, we characterized the antimicrobial activity of the peptoid **D2** against the canine pathogen *S. pseudintermedius*. We optimized its activity via a structure–activity study, involving Gly-scan single residue substitutions, exploring the effect of the peptoid backbone by converting **D2** to the corresponding peptides. The most potent analog, **D2D**, containing all d-amino acids, showed excellent killing kinetics against MRSP and activity against a panel of canine pathogens. Fluorescence spectroscopy and a macromolecule biosynthesis assay revealed that **D2D** at sub-MIC concentrations inhibited cell-wall synthesis, rather than disrupted bacterial membrane. This study confirms the potential of peptides and peptidomimetics as veterinary antimicrobial agents, and the crucial role of backbone nature in the discovery process of peptide-based therapeutics.

## Figures and Tables

**Table 1:** Structures, antimicrobial activity (MIC, µg/mL) and hemolytic activity (% at 150 µM) of eight analogues of **D2** resulting from Gly-scan.

ID	SEQUENCE ^a^								MIC				HA ^f^
									MSSP ^b^	MRSP ^c^	SA ^d^	PA ^e^	
**D2**	Nlys	Nlys	N1Nal	N4MePhe	Nlys	N1Nal	Nlys	NNle	2–4	4	8	16	24
**1**	Gly	Nlys	N1Nal	N4MePhe	Nlys	N1Nal	Nlys	NNle	4	4	16	32	42
**2**	Nlys	Gly	N1Nal	N4MePhe	Nlys	N1Nal	Nlys	NNle	16	8	16	64	48
**3**	Nlys	Nlys	Gly	N4MePhe	Nlys	N1Nal	Nlys	NNle	64	64	>64	>64	56
**4**	Nlys	Nlys	N1Nal	Gly	Nlys	N1Nal	Nlys	NNle	64	32	>64	>64	40
**5**	Nlys	Nlys	N1Nal	N4MePhe	Gly	N1Nal	Nlys	NNle	4	4	32	16	25
**6**	Nlys	Nlys	N1Nal	N4MePhe	Nlys	Gly	Nlys	NNle	>64	>64	>64	>64	6
**7**	Nlys	Nlys	N1Nal	N4MePhe	Nlys	N1Nal	Gly	NNle	8	4	16	16	85
**8**	Nlys	Nlys	N1Nal	N4MePhe	Nlys	N1Nal	Nlys	Gly	8	8	32	32–64	49

^a^ Products synthesized as C-terminal amides; ^b^ Methicillin sensitive *Staphylococcus pseudintermedius* (26916); ^c^ Methicillin resistant *Staphylococcus pseudintermedius*, (C22963); ^d^ *Staphylococcus aureus* (ATCC 29213); ^e^ *Pseudomonas aeruginosa* (ATCC 27853); ^f^ Percentage of hemolysis at 150 µM.

**Table 2:** Effect of single substitutions in position 5 on antibacterial (MIC, µg/mL) and hemolytic activity (% at 150 µM) of **D2**.

ID	SEQUENCE ^a^								MIC				HA ^f^
									MSSP ^b^	MRSP ^c^	SA ^d^	PA ^e^	
**D2**	Nlys	Nlys	N1Nal	N4MePhe	Nlys	N1Nal	Nlys	NNle	2–4	4	8	16	24
**9**					NMe				4	4	16	32	16
**10**	N4MePhe								8	8	8	16	69
**11**	N1Nal								8	8	8	16	87
**12**	NEtOH								8	8	32	>64	5

^a^ Products synthesized as C-terminal amides; ^b^ Methicillin sensitive *Staphylococcus pseudintermedius* (26916); ^c^ Methicillin resistant *Staphylococcus pseudintermedius*, (C22963); ^d^ *Staphylococcus aureus* (ATCC 29213); ^e^ *Pseudomonas aeruginosa* (ATCC 27853); ^f^ Percentage of hemolysis at 150 µM.

**Table 3:** Structures, antimicrobial (MIC, µg/mL), and hemolytic activity (% at 150 µM) of l- (**D2L**), d- (**D2D**), retro- (**D2R**), and peptide–peptoid hybrid analogues (**13**–**16**) of **D2**.

ID	SEQUENCE ^a^								MIC				HA ^f^
									MSSP ^b^	MRSP ^c^	SA ^d^	PA ^e^	
**D2**	Nlys	Nlys	N1Nal	N4MePhe	Nlys	N1Nal	Nlys	NNle	2–4	4	8	16	24
**D2L**	Lys	Lys	1Nal	Phe	Lys	1Nal	Lys	Nle	16	8	32	16	35
**D2D**	lys	lys	1nal	phe	lys	1nal	lys	nle	1–4	4	4	4	35
**D2R**	Nle	Lys	1Nal	Lys	Phe	1Nal	Lys	Lys	16	4	32	32–64	23
**13**	Nlys	Nlys	N1Nal	N4MePhe	lys	1nal	lys	nle	4	4	8	8	34
**14**	lys	lys	1nal	phe	Nlys	N1Nal	Nlys	NNle	4	4	16	64	5
**15**	Nlys	Nlys	1nal	phe	Nlys	1nal	Nlys	nle	4	4	32	>64	6
**16**	lys	lys	N1Nal	N4MePhe	lys	N1Nal	lys	NNle	4	4	16	64	4

^a^ Products synthesized as C-terminal amides; ^b^ Methicillin sensitive *Staphylococcus pseudintermedius* (26916); ^c^ Methicillin resistant *Staphylococcus pseudintermedius*, (C22963); ^d^ *Staphylococcus aureus* (ATCC 29213); ^e^ *Pseudomonas aeruginosa* (ATCC 27853); ^f^ Percentage of hemolysis at 150 µM.

**Table 4:** Spectrum of antimicrobial activity of **D2** and **D2D** against a broad range of canine isolates, including Gram-positive and Gram-negative species.

Bacteria	D2	D2D
*Acinetobacter baumannii*, 27065, 16 D1, dog, wound, 2010	>64	16
*Corynebacterium auriscanis*, 31551, 54 C6, dog, ear, 2013	2	2
*E. coli*, 30235, 23 A6, dog, wound, 2012	64	4
*Enterococcus faecalis*, 27404, 17 C7, dog, wound, 2011	>64	8
*Enterococcus faecium*, 30951, 24 C1, dog, ear, 2013	16	4
*Klebsiella pneumoniae*, 26233, 11 H5, dog, wound, 2010	>64	8
*Proteus mirabilis*, 25178, 9 A4, dog, ear, 2009	>64	>64
*Pseudomonas aeruginosa* (26314, 12 C5, dog, urine, 2010)	32	8

## Supplementary Materials

The following are available online, Figure S1: Analytical HPLC Chromatograms after Purifications; Figure S2: MIC Distribution; Figure S3: Growth Curves; Figure S4: Growth Curves; Table S1: Table with Compounds, Mass, and HPLC Retention Times; Table S2: MIC selectivity of **D2** and **D2D** between *S. aureus* and *S. intermedius*.
