# Supplementary material for: Structure–Activity Study, Characterization, and Mechanism of Action of an Antimicrobial Peptoid D2 and Its d- and l-Peptide Analogues
Source: Molecules. 2019 Mar 21;24(6):1121. doi: 10.3390/molecules24061121 (PMC6470533; doi:10.3390/molecules24061121)
Supplement: Supplementary file 1 [file molecules-24-01121-s001.pdf]

molecules

MDPI

Structure–Activity Study, Characterization, and Mechanism of Action of an Antimicrobial Peptoid D2 and Its D- and L-Peptide Analogues

Ines Greco [1,†], Johannes E. Hansen [1], Bimal Jana [2], Natalia Molchanova[1,‡], Alberto Oddo [1,§], Peter W. Thulstrup [3], Peter Damborg [2], Luca Guardabassi [2,4], and Paul R. Hansen [1,*]

[1] Department of Drug Design and Pharmacology, Faculty of Health and Medical Sciences, University of Copenhagen, Universitetsparken 2, 2100, Copenhagen, Denmark; ines.greco@food.ku.dk (I.G.); johanneselton@hotmail.com (J.E.H.); natalia.molchanova@sund.ku.dk (N.M.); albi.oddo@gmail.com (A.O.)

[2] Department of Veterinary and Animal Sciences, Faculty of Health and Medical Sciences, University of Copenhagen, Stigbøjlen 4, 1870, Frederiksberg C, Denmark; bimal@sund.ku.dk (B.J.); pedam@sund.ku.dk (P.D.); lg@sund.ku.dk (L.G.)

[3] Department of Chemistry, University of Copenhagen, Universitetsparken 5, 2100 Copenhagen, Denmark; pwt@chem.ku.dk

[4] Department of Pathobiology and Population Sciences, The Royal Veterinary College, Hawkshead Lane, North Mymms, Hatfield, Herts, AL9 7TA, UK

* Correspondence: prh@sund.ku.dk; Tel.: +4535336625

† Present address: Department of Food Science, Faculty of Science, University of Copenhagen, Rolighedsvej 30, 1958, Frederiksberg, Denmark

‡ Present address: Department of Science and Environment, Roskilde University, Universitetsvej 1, 4000 Roskilde, Denmark

§ Present address: Novo Nordisk A/S, Krogshøjvej 44, 2820 Bagsværd, Denmark

Received: 18 February 2019; Accepted: 12 March 2019; Published: 21 March 2019

TABLE OF CONTENTS:

Table S1: Table With Compounds; Mass and HPLC Retention Times...2

Figure S1: Analytical HPLC Chromatograms after purifications.. 5

Figure S2: MIC distribution (μM) for 100 isolates tested against the compound D2...............................15

Figure S3: Growth Curves for (a) D2; (b) D2-D; (c) D2L; (d) D2R and (e) nisin.......................................15

TABLE S2: MIC selectivity of D2 and D2D between *S. aureus* and *S. intermedius*..................................16

Figure S4 CD SPECTRA OF D2D, D2L, D2R, 13, 14, 15 and 16..17

Table S1: TABLE WITH COMPOUNDS; MASS AND HPLC RETENTION TIMES

ID	STRUCTURE	Mass Calc´d	Mass Obs´d	HPLC RT/min
**D2**	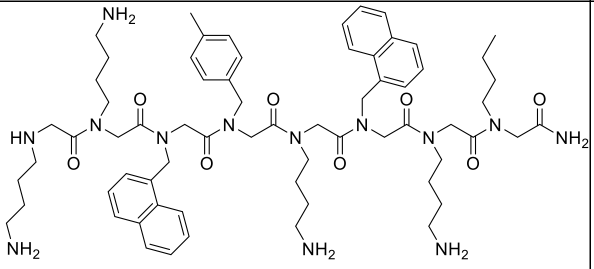	1198.6	1998.6	16.2
**1**	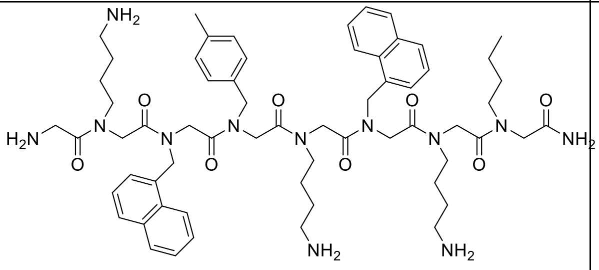	1127.5	1127.4	17.1
**2**	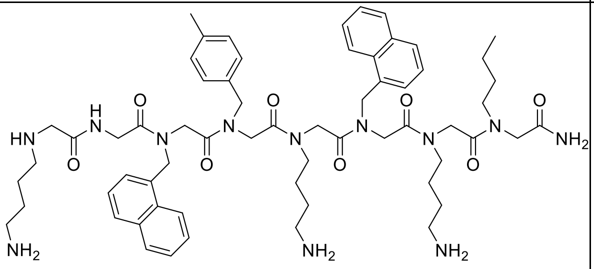	1127.5	1128.2	17.4
**3**	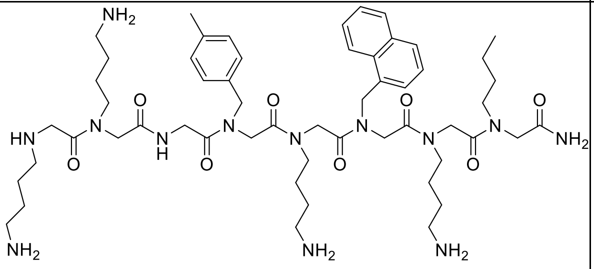	1058.4	1058.6	14.8
**4**	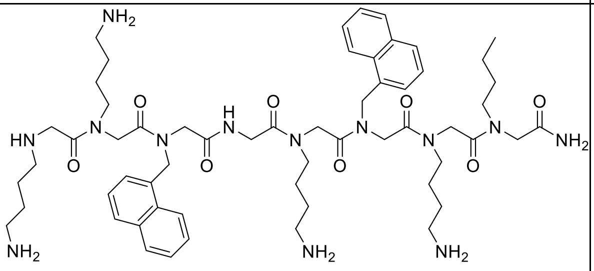	1094.4	1094.6	14.9

**5**	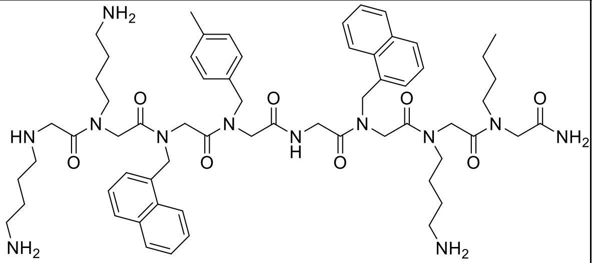	1127.5	1127.6	17.2
**6**	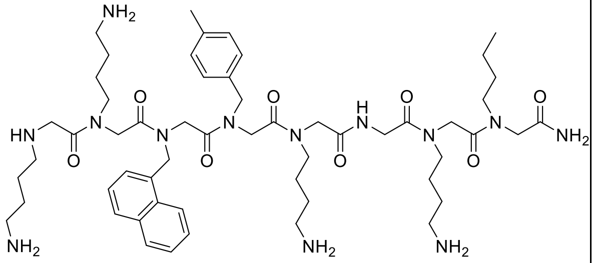	1058.4	1058.6	14.6
**7**	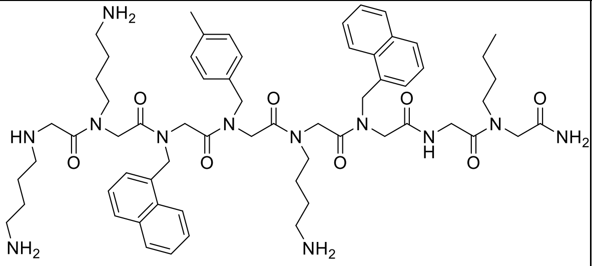	1127.5	1127.6	17.6
**8**	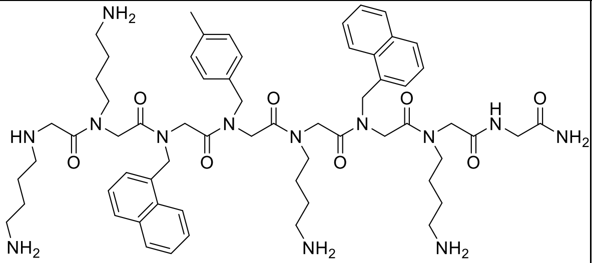	1142.5	1142.6	15.6
**9**	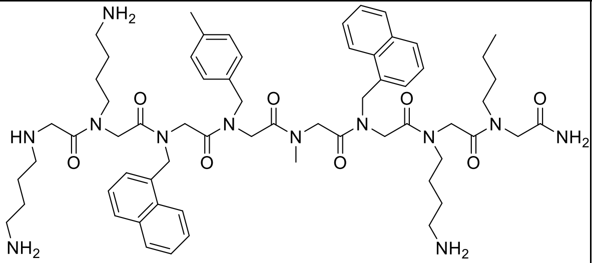	1141.5	1141.4	17.4
**10**	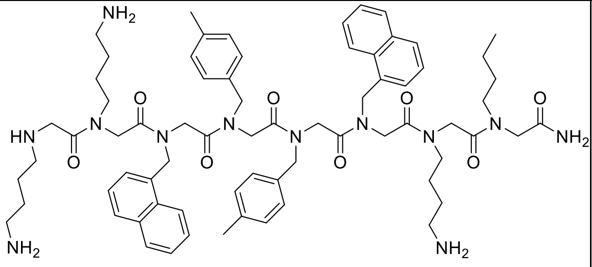	1231.6	1231.6	18.7

**11**	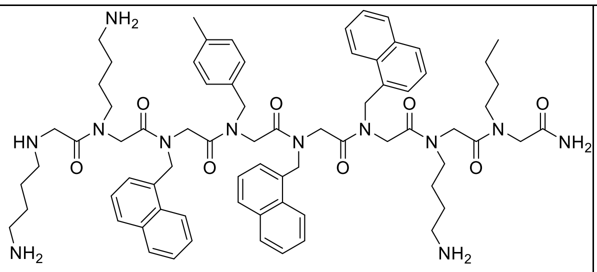	1267.6	1267.8	19.1
**12**	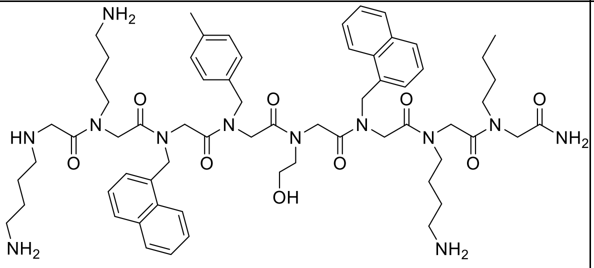	1171.5	1171.6	17.1
**D2L**	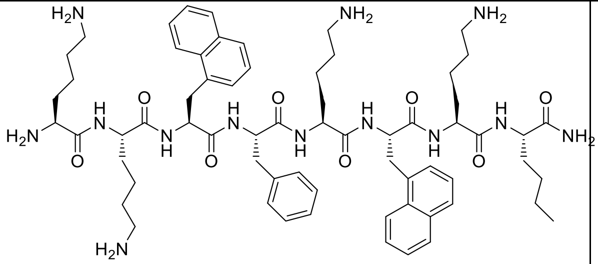	1184.5	1184.5	15.5
**D2D**	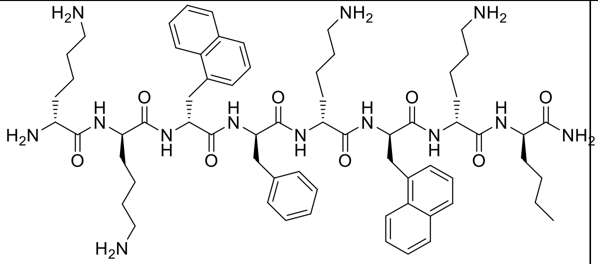	1184.5	1184.7	15.6
**D2R**	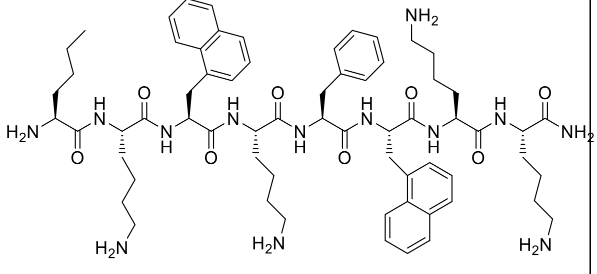	1184.5	1184.7	15.4

**13**	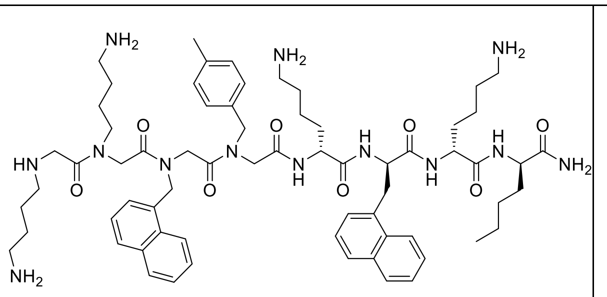	1198.6	1198.6	16.4
**14**	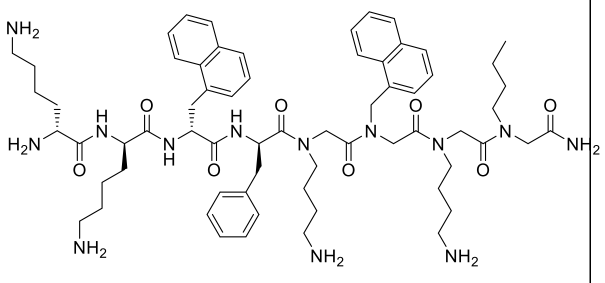	1184.5	1184.7	16.4
**15**	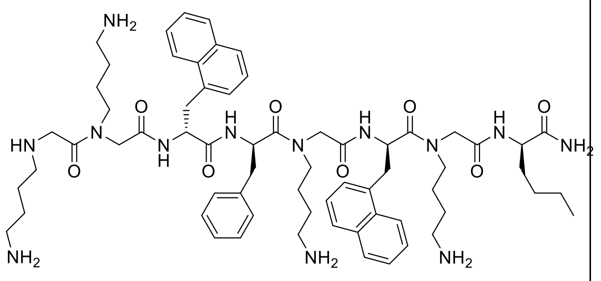	1184.5	1184.5	15.3
**16**	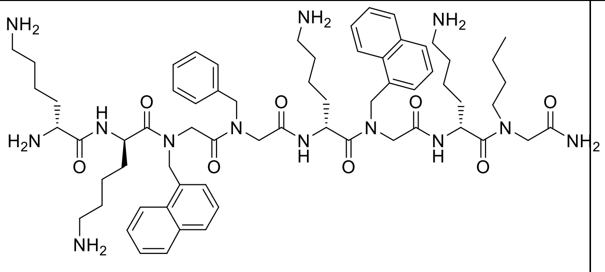	1198.6	1198.9	15.5

Figure S1 Analytical HPLC Chromatograms after purifications

D2:

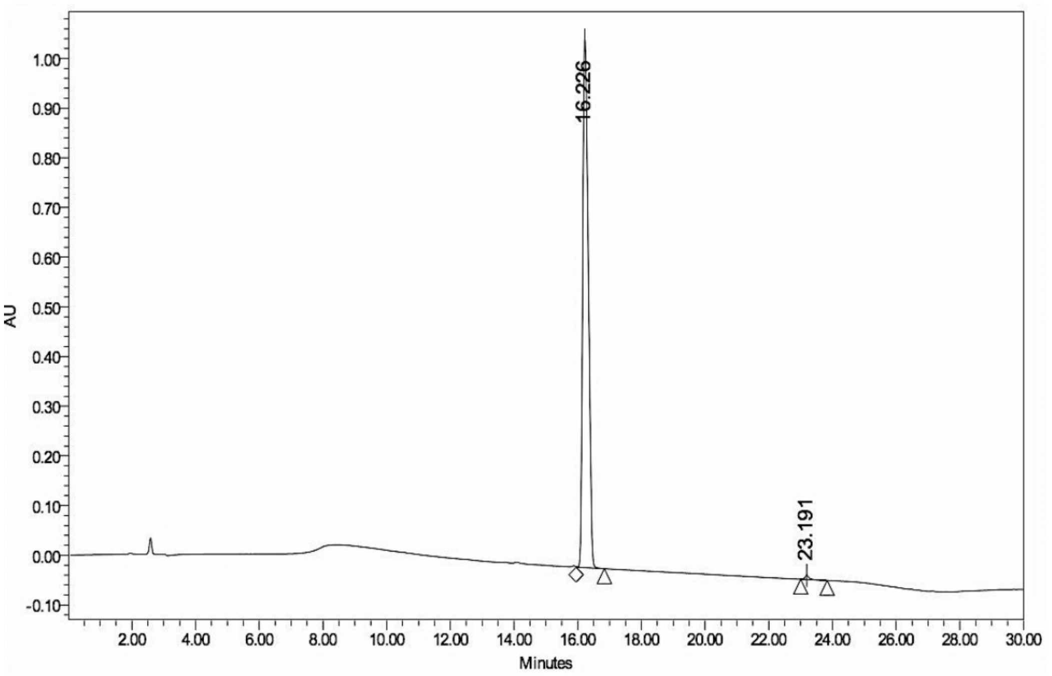
16.226
23.191
AU
1.00
0.90
0.80
0.70
0.60
0.50
0.40
0.30
0.20
0.10
0.00
-0.10
2.00
4.00
6.00
8.00
10.00
12.00
14.00
16.00
18.00
20.00
22.00
24.00
26.00
28.00
30.00
Minutes

D2L:

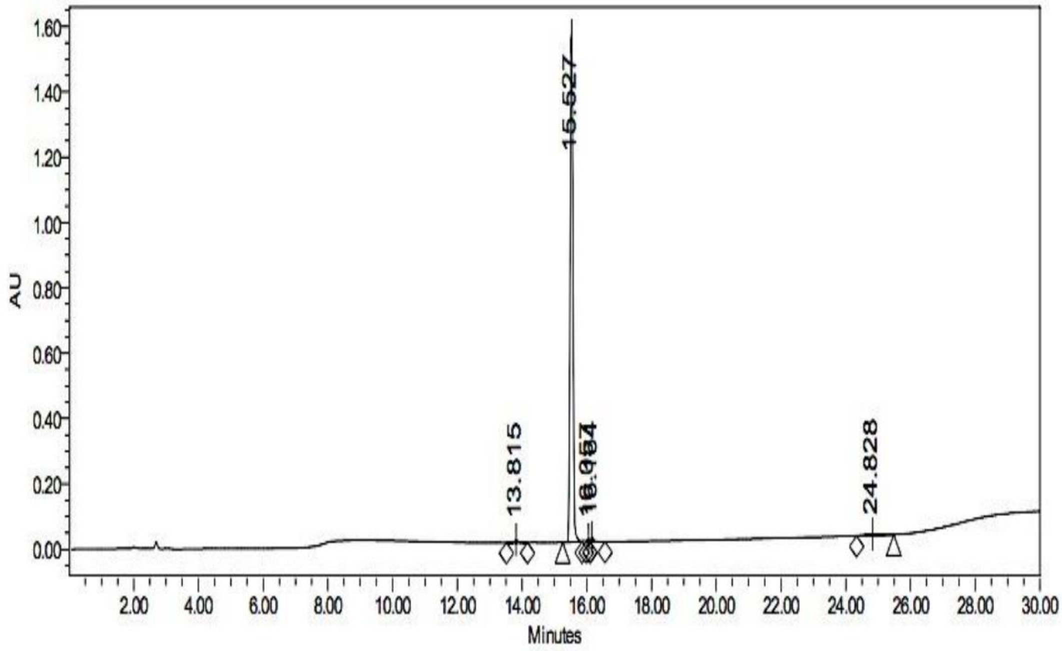
15.527
13.815
24.828
AU
1.60
1.40
1.20
1.00
0.80
0.60
0.40
0.20
0.00
2.00
4.00
6.00
8.00
10.00
12.00
14.00
16.00
18.00
20.00
22.00
24.00
26.00
28.00
30.00
Minutes

D2D:

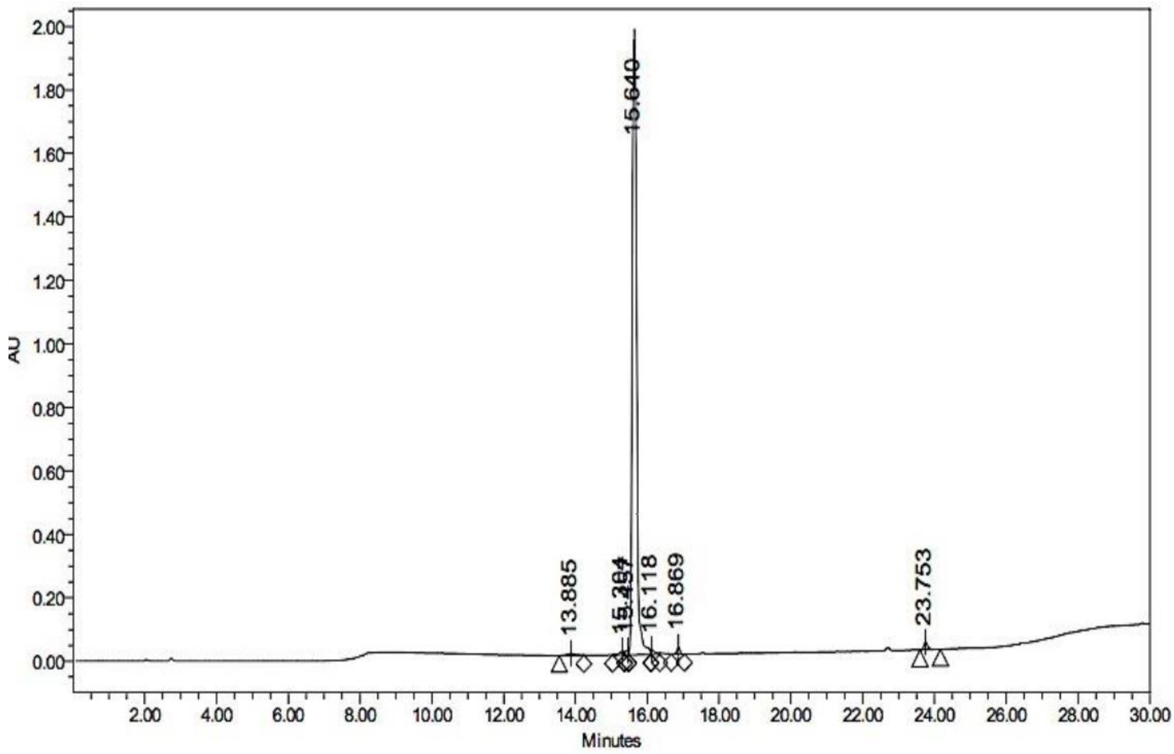

15.640
13.885
15.304
15.457
16.118
16.869
23.753
AU
Minutes

D2R:

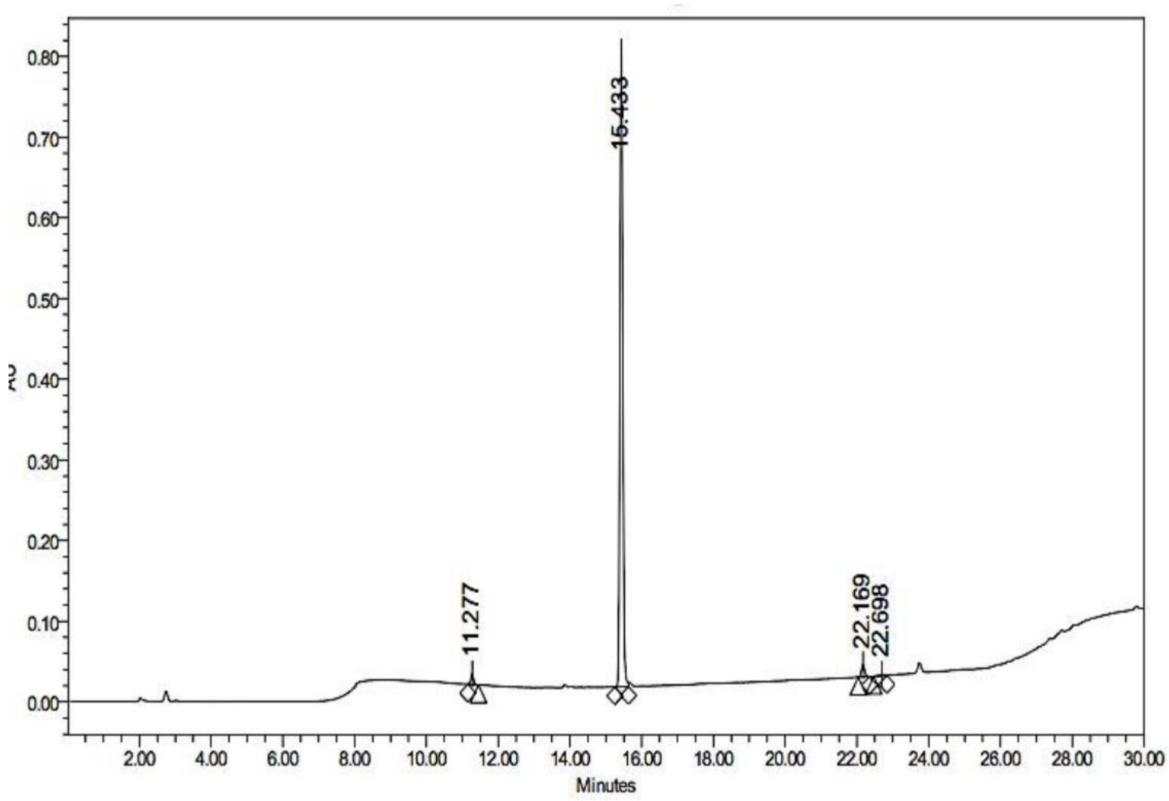

15.433
11.277
22.169
22.698
Minutes

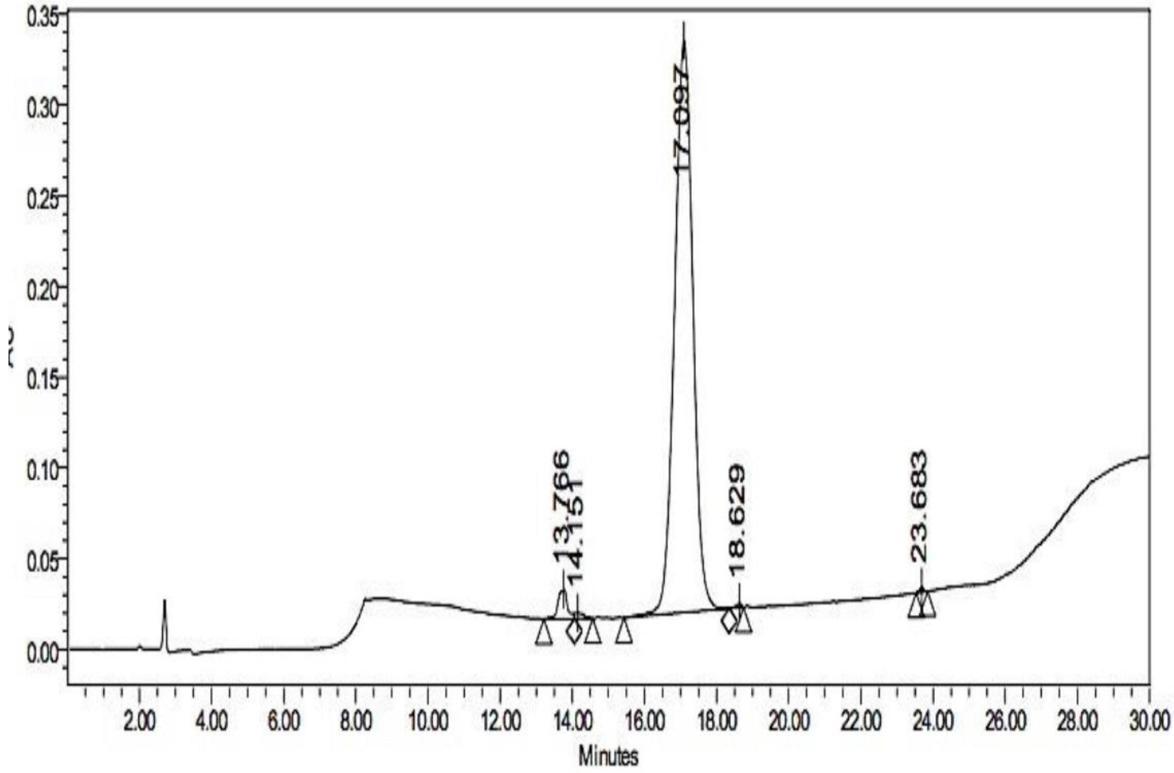
0.35
0.30
0.25
0.20
0.15
0.10
0.05
0.00
17.097
13.766
14.151
18.629
23.683
2.00 4.00 6.00 8.00 10.00 12.00 14.00 16.00 18.00 20.00 22.00 24.00 26.00 28.00 30.00
Minutes

2

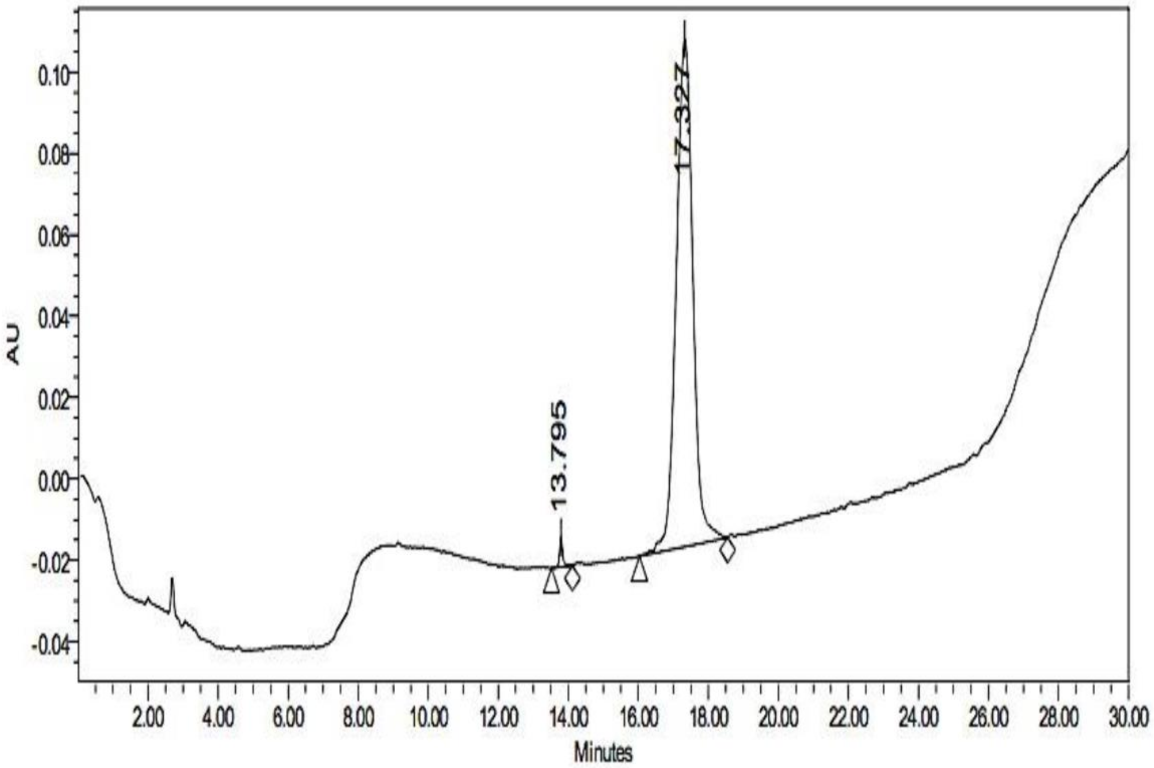
AU
0.10
0.08
0.06
0.04
0.02
0.00
-0.02
-0.04
17.327
13.795
2.00 4.00 6.00 8.00 10.00 12.00 14.00 16.00 18.00 20.00 22.00 24.00 26.00 28.00 30.00
Minutes

3

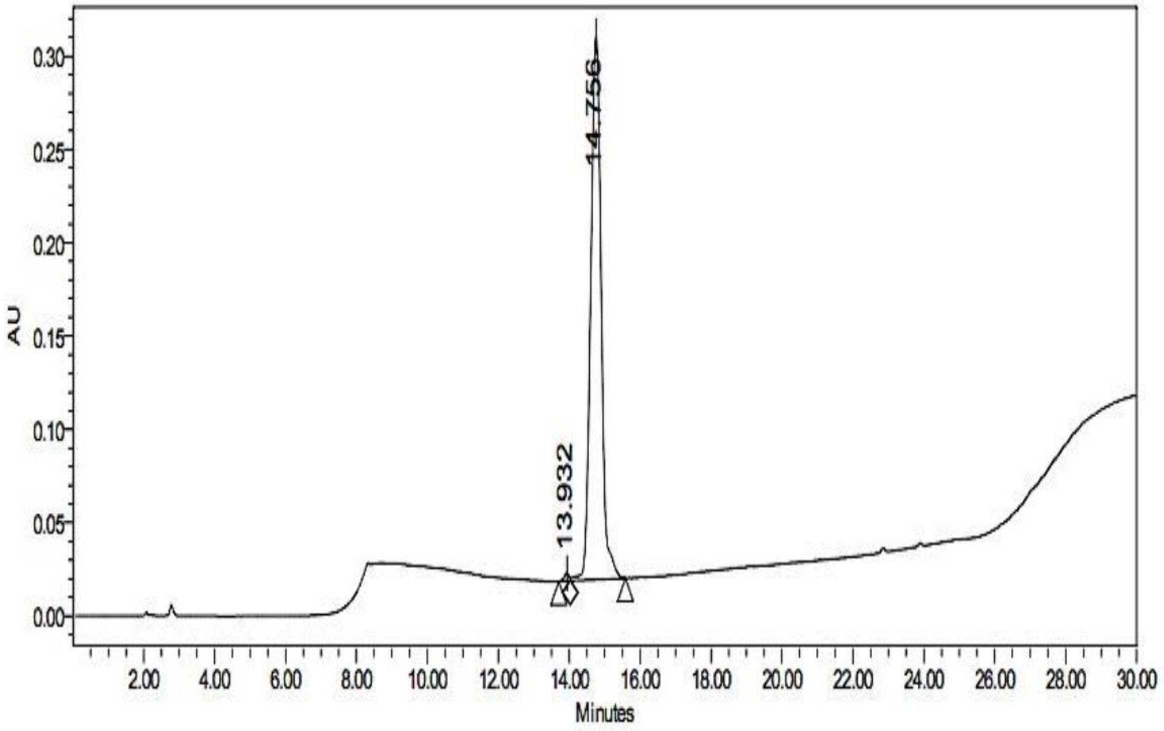
14.756
13.932
AU
Minutes
0.30
0.25
0.20
0.15
0.10
0.05
0.00
2.00
4.00
6.00
8.00
10.00
12.00
14.00
16.00
18.00
20.00
22.00
24.00
26.00
28.00
30.00

4

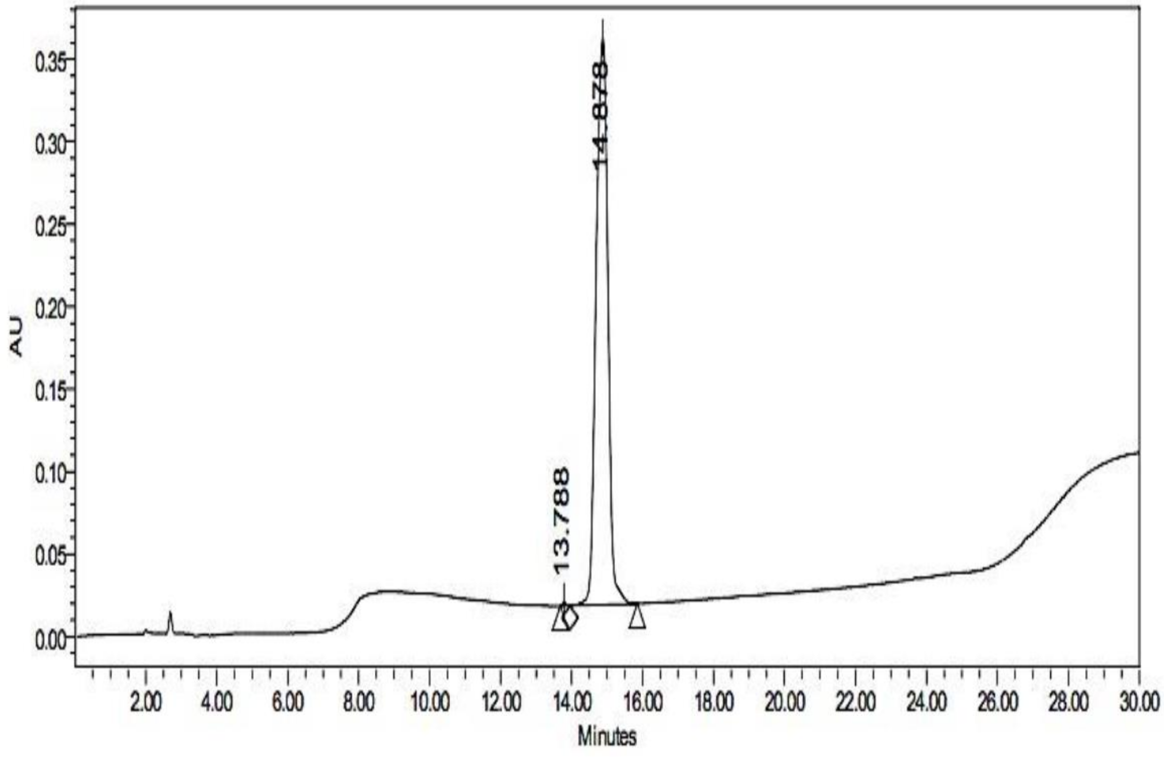
14.878
13.788
AU
Minutes
0.35
0.30
0.25
0.20
0.15
0.10
0.05
0.00
2.00
4.00
6.00
8.00
10.00
12.00
14.00
16.00
18.00
20.00
22.00
24.00
26.00
28.00
30.00

5

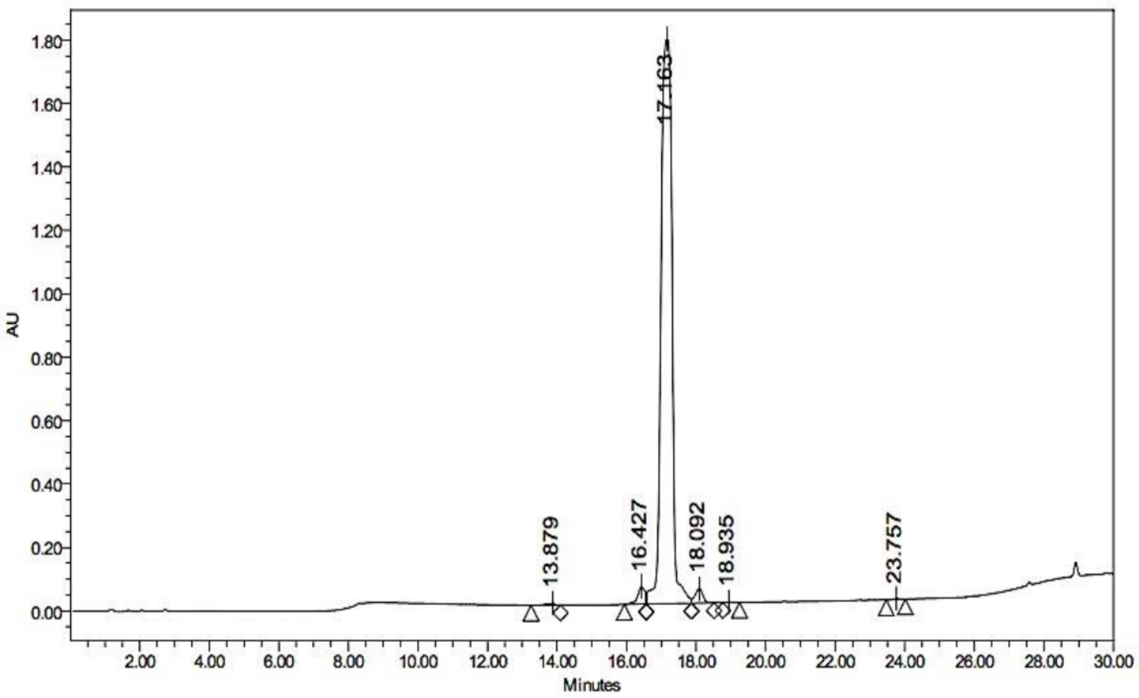

17.163
16.427
13.879
18.092
18.935
23.757
AU
Minutes

6

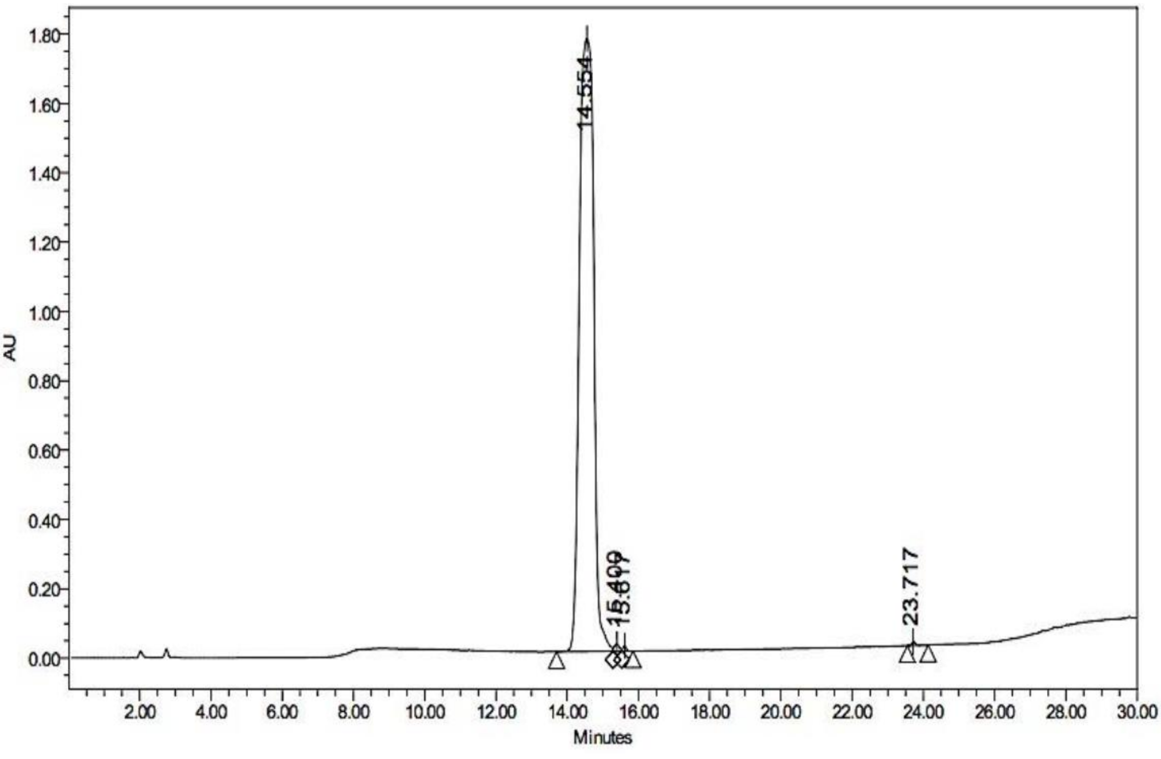

14.554
15.409
15.617
23.717
AU
Minutes

7

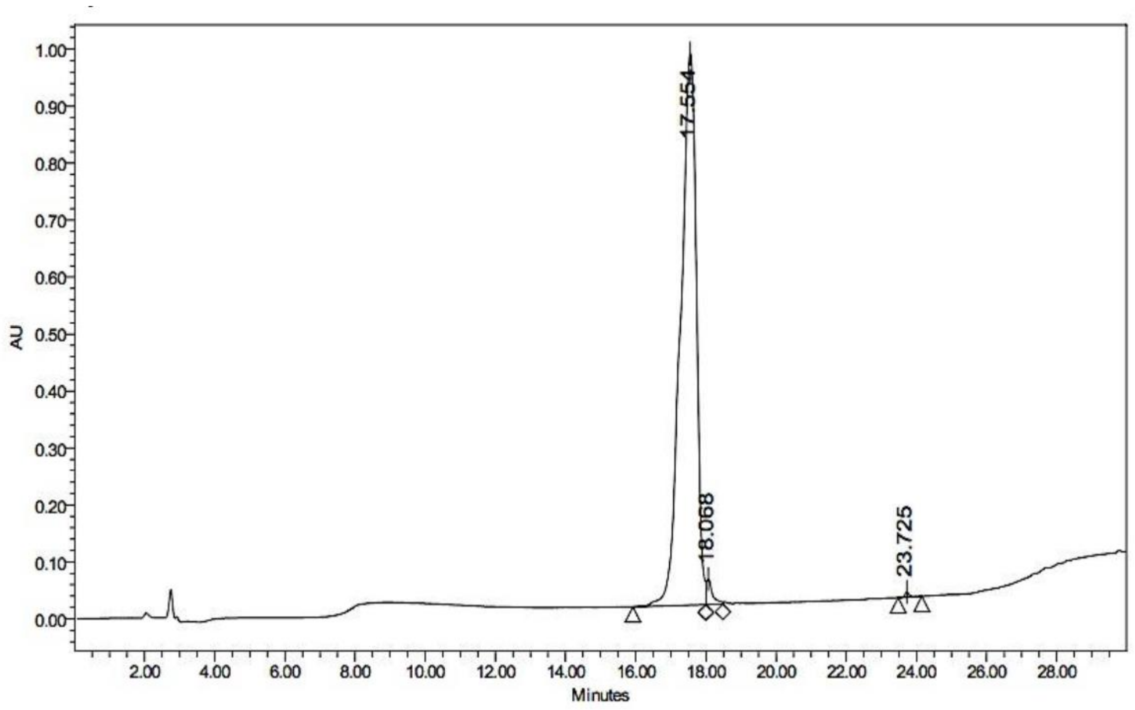

1.00
0.90
0.80
0.70
0.60
0.50
0.40
0.30
0.20
0.10
0.00
AU
17.554
18.068
23.725
2.00
4.00
6.00
8.00
10.00
12.00
14.00
16.00
18.00
20.00
22.00
24.00
26.00
28.00
Minutes

8

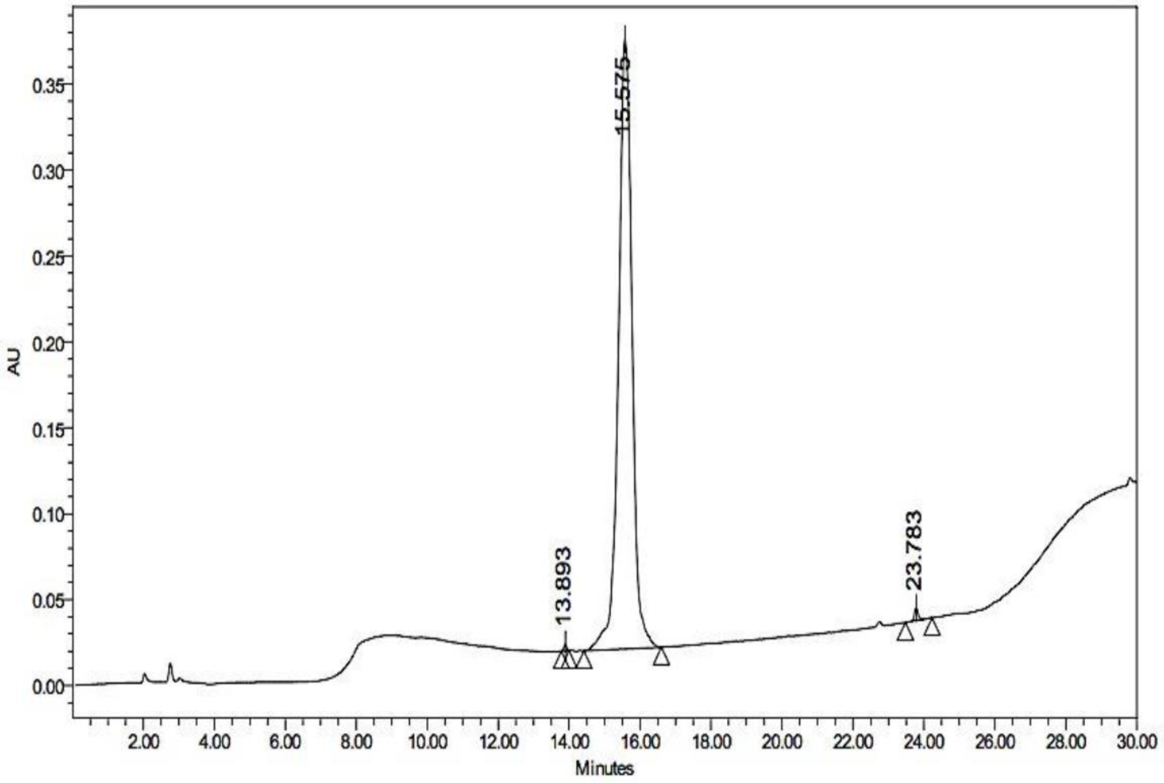

0.35
0.30
0.25
0.20
0.15
0.10
0.05
0.00
AU
15.575
13.893
23.783
2.00
4.00
6.00
8.00
10.00
12.00
14.00
16.00
18.00
20.00
22.00
24.00
26.00
28.00
30.00
Minutes

9

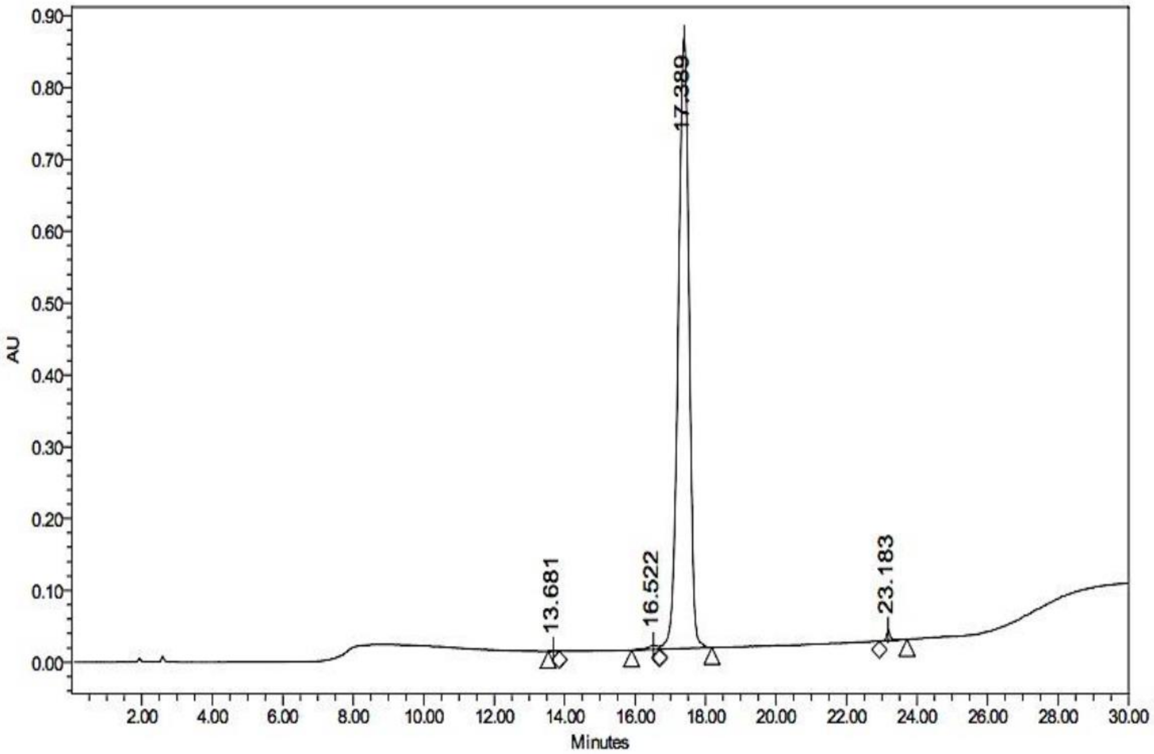
AU
Minutes
17.389
13.681
16.522
23.183
0.90
0.80
0.70
0.60
0.50
0.40
0.30
0.20
0.10
0.00
2.00
4.00
6.00
8.00
10.00
12.00
14.00
16.00
18.00
20.00
22.00
24.00
26.00
28.00
30.00

10

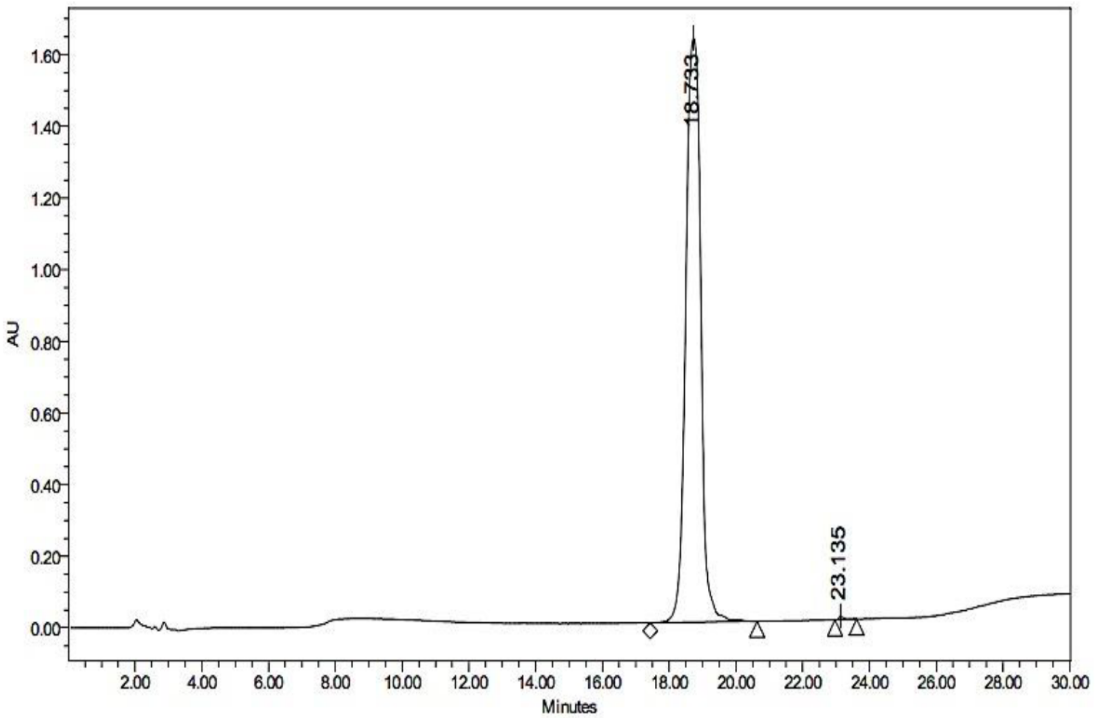
AU
Minutes
18.733
23.135
1.60
1.40
1.20
1.00
0.80
0.60
0.40
0.20
0.00
2.00
4.00
6.00
8.00
10.00
12.00
14.00
16.00
18.00
20.00
22.00
24.00
26.00
28.00
30.00

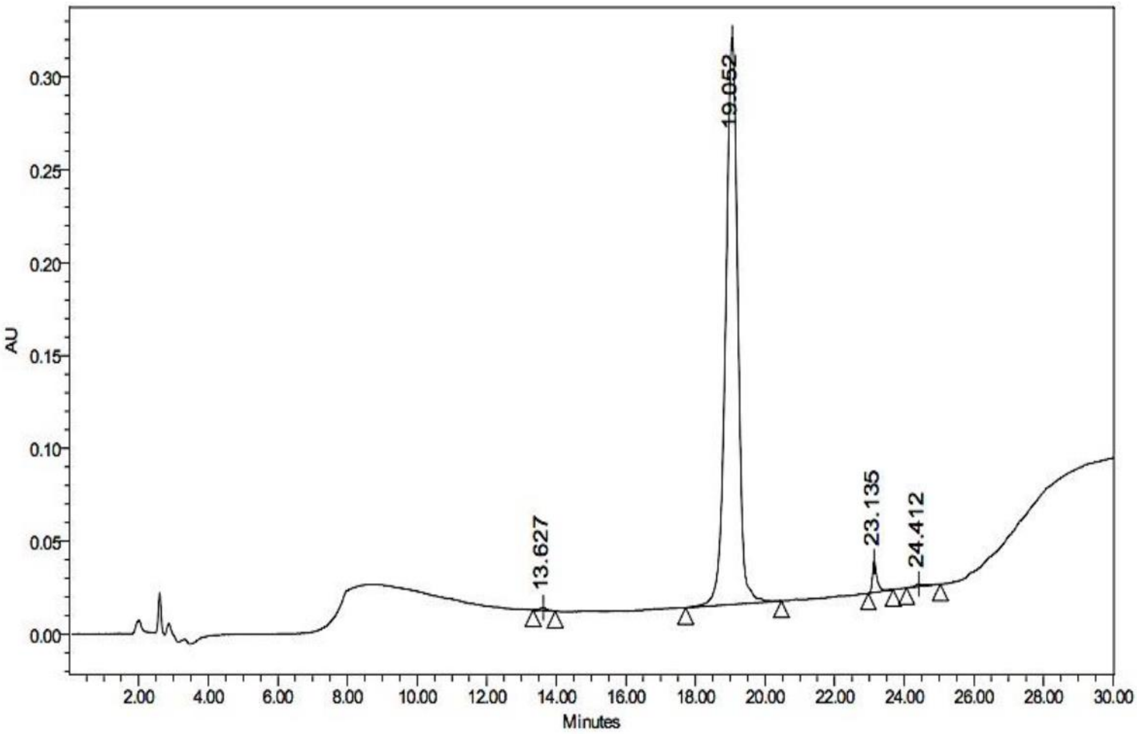
19.052
13.627
23.135
24.412
AU
0.30
0.25
0.20
0.15
0.10
0.05
0.00
2.00 4.00 6.00 8.00 10.00 12.00 14.00 16.00 18.00 20.00 22.00 24.00 26.00 28.00 30.00
Minutes

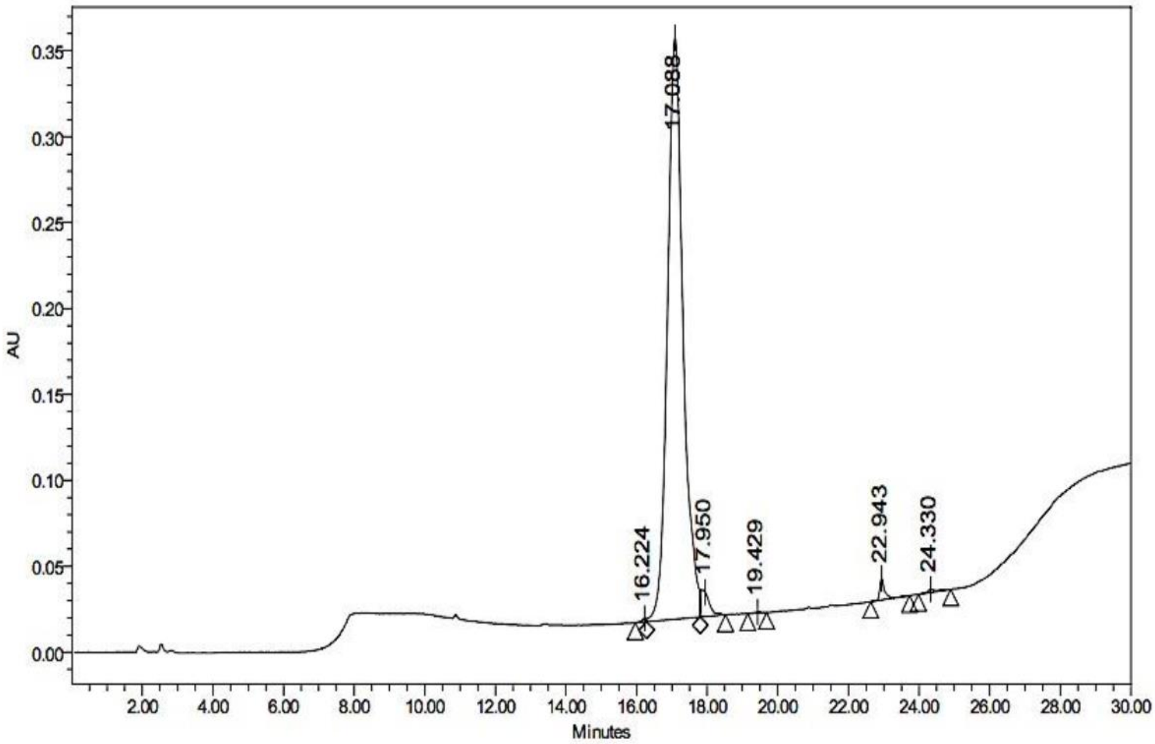
17.088
16.224
17.950
19.429
22.943
24.330
AU
0.35
0.30
0.25
0.20
0.15
0.10
0.05
0.00
2.00 4.00 6.00 8.00 10.00 12.00 14.00 16.00 18.00 20.00 22.00 24.00 26.00 28.00 30.00
Minutes

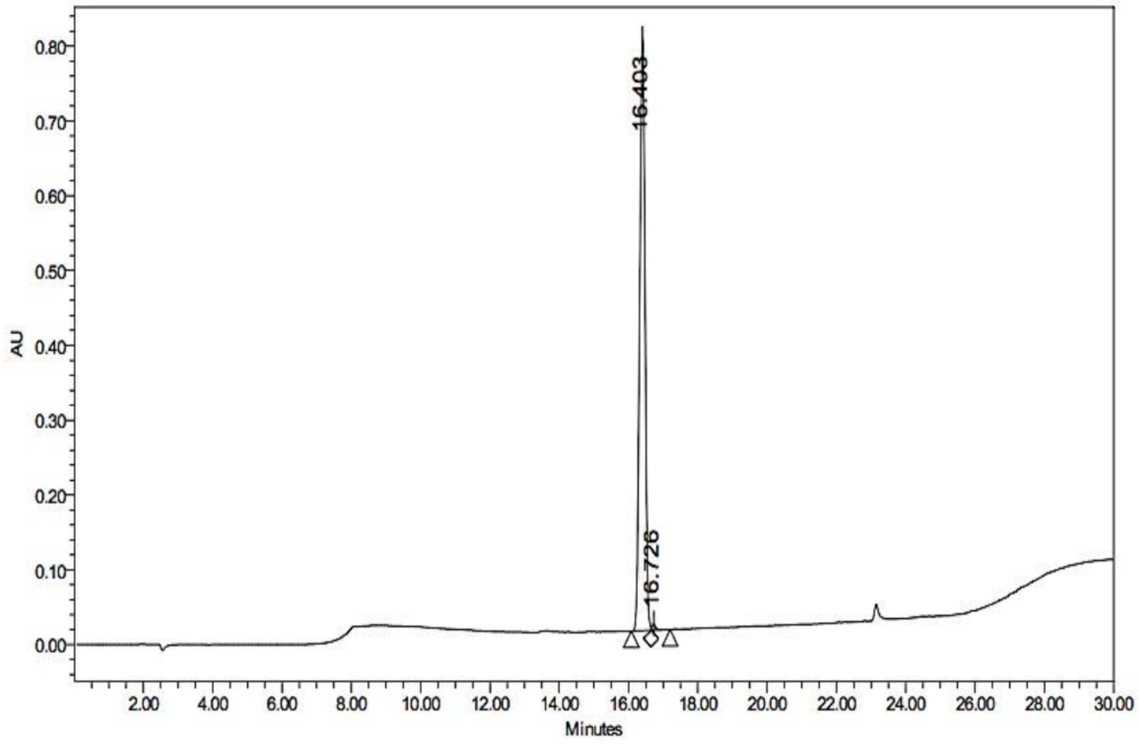

16.403
16.726
AU
Minutes
0.00
0.10
0.20
0.30
0.40
0.50
0.60
0.70
0.80
2.00
4.00
6.00
8.00
10.00
12.00
14.00
16.00
18.00
20.00
22.00
24.00
26.00
28.00
30.00

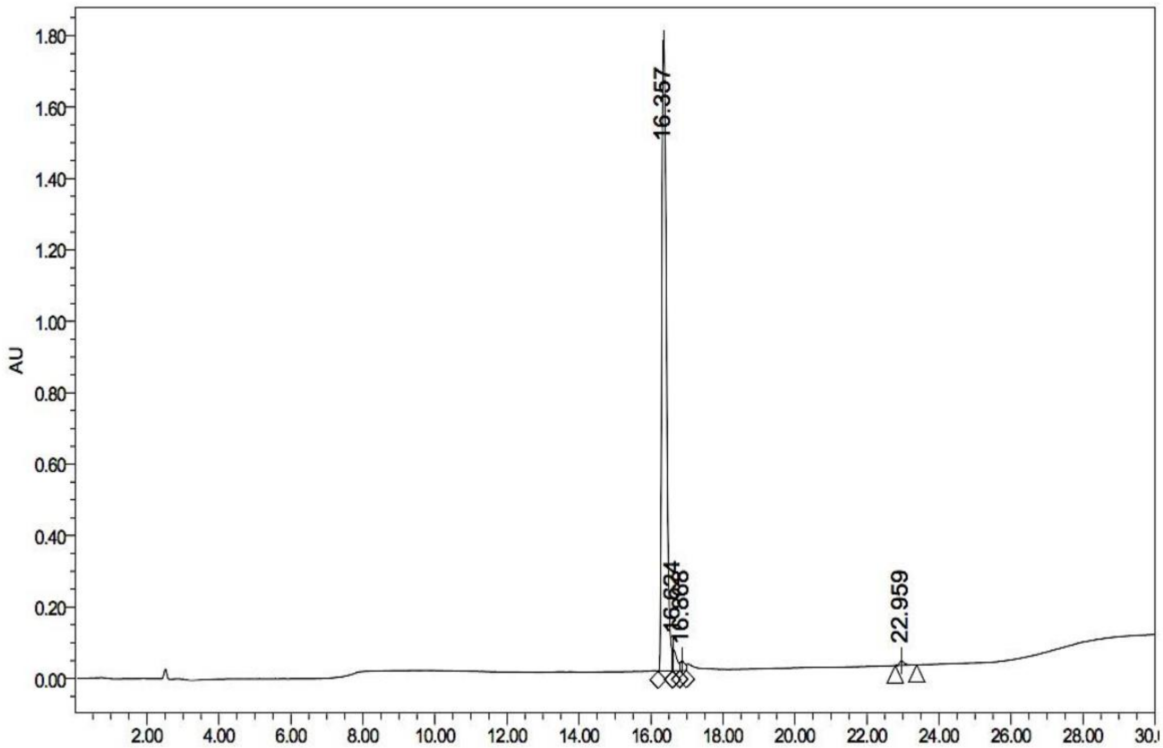

16.357
22.959
AU
0.00
0.20
0.40
0.60
0.80
1.00
1.20
1.40
1.60
1.80
2.00
4.00
6.00
8.00
10.00
12.00
14.00
16.00
18.00
20.00
22.00
24.00
26.00
28.00

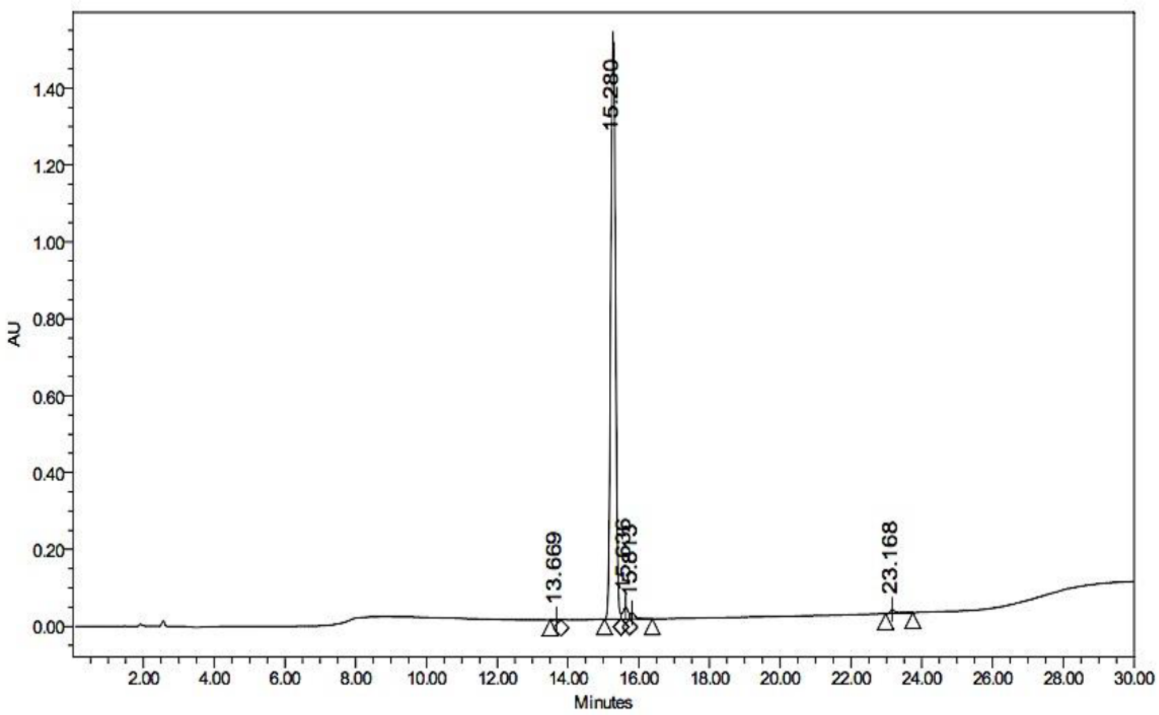

16

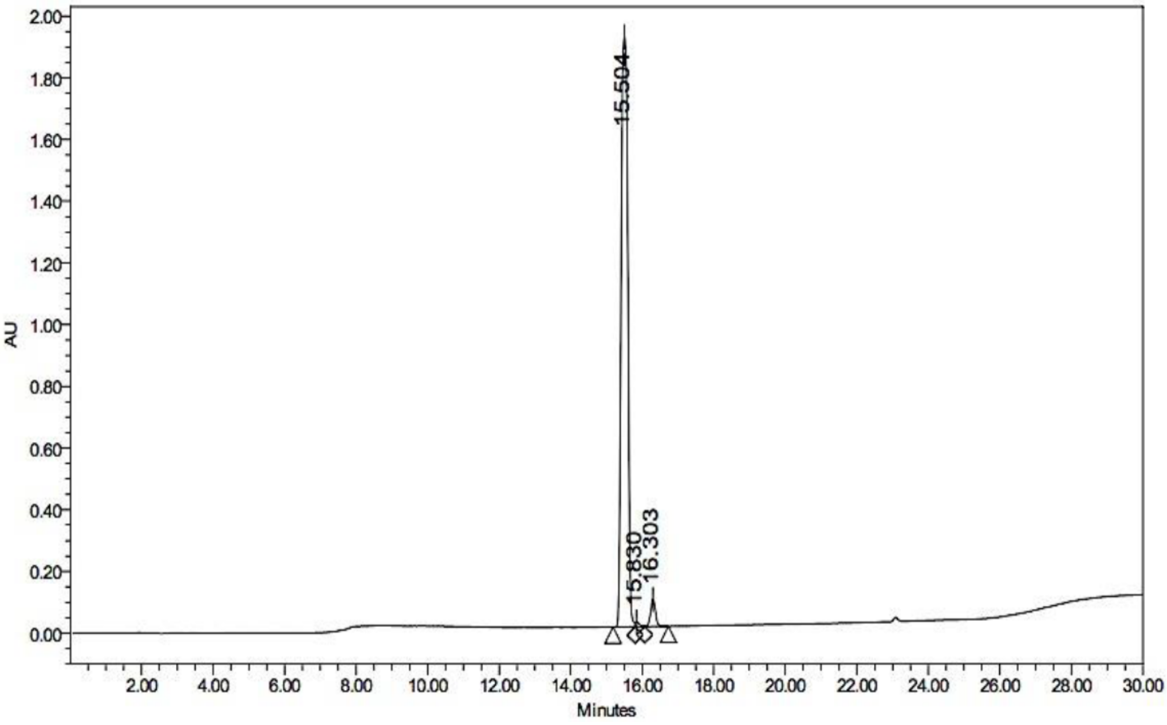

Figure S2 MIC distribution (μM) for 100 isolates tested against the compound D2

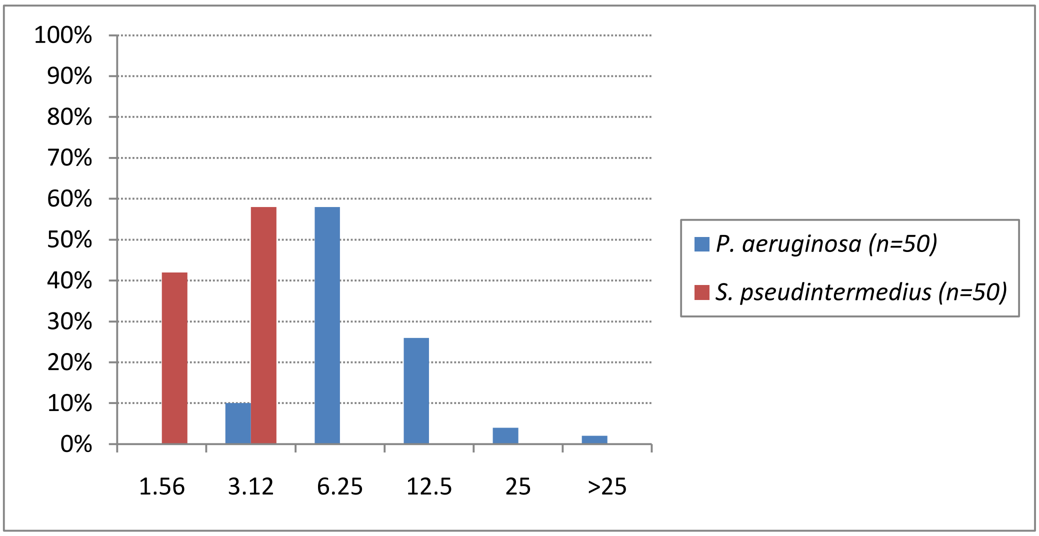

Figure S3. Growth Curves for (a) D2; (b) D2-D; (c) D2L; (d) D2R and (e) nisin

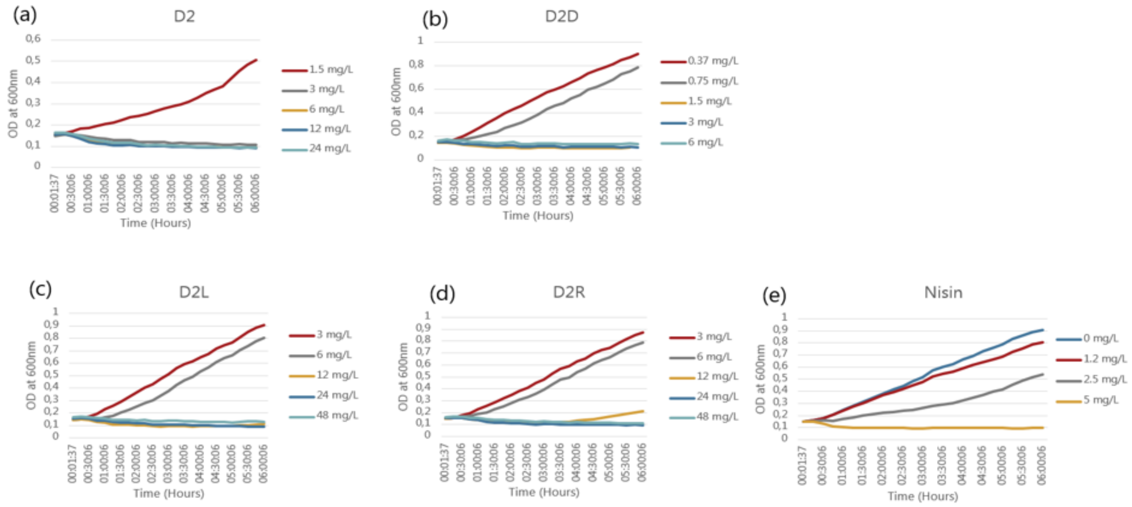

TABLE S2: MIC selectivity of D2 and D2D between *S. aureus* and *S. intermedius*.

	D2	D2D
S. aureus		
25054, 8 G6, dog, wound, 2009	4	2
27266, 16 G9, dog, skin, 2010	8	2
28264, 20 B1, dog, wound, 2011	4	4
30935, 24 B9, dog, joint, 2013	4	2
36968, 61 A9, dog, wound, 2016	4	4
37595, 65 D2, dog, joint, 2016	4	2
37708-2, 66 C6, dog, skin, 2016	8	4
38200, 68 E9, dog, skin, 2016	4	2
38565-1, 70 A5, dog, skin, 2017	4	2
38841, 70 G5, dog, urine, 2017	8	4
S. pseudintermedius		
22963, 3 B9, dog, ?, 2007	1-2	2
26071, 11 E5, dog, skin, 2009	1	1
26092-2, 11 E4, dog, skin, 2009	1	1
26959, 15 F8, dog, wound, 2010	2	2
27364, 17 A7, dog, wound, 2011	1	2
27382, 17 B8, dog, ear, 2011	1	2
31524, 54 C3, dog, ear, 2013	1	2
33228, 55 E2, dog, skin, 2014	1	2
35890, 59 B8, dog, ear, 2015	1	2
37526-1, 65 B4, dog, skin, 2016	1	2
37535-1, 65 B6, dog, skin, 2016	1	2
38637, 70 C3, dog, wound 2017	1	2
38820, 70 F9, dog, urine 2017	1	2

Figure S4 CD SPECTRA OF D2D, D2L, D2R, 13, 14, 15 and 16

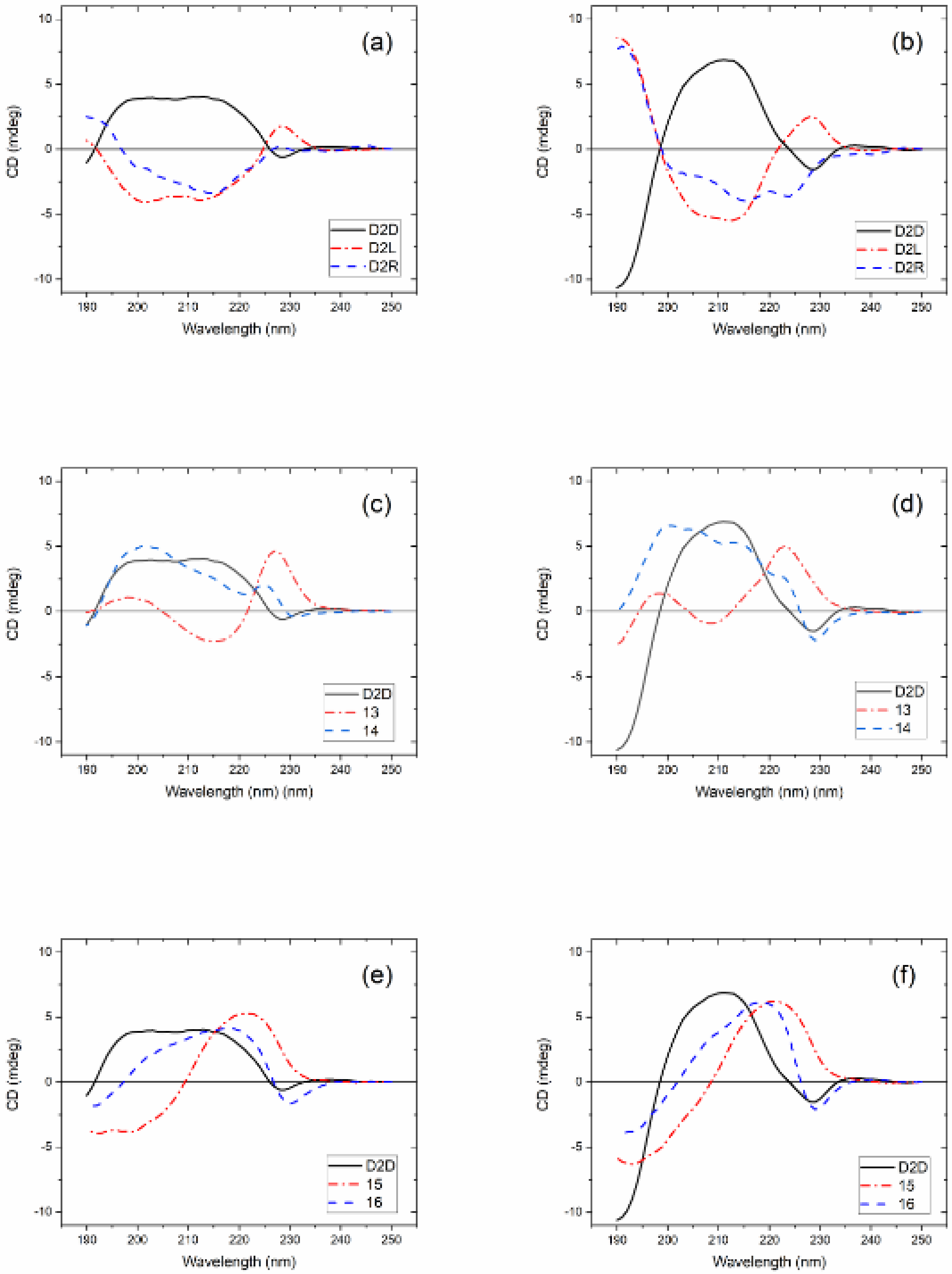

Figure S4. Circular dichroism (CD) spectra in the far-UV range of selected peptide and mixed peptiod sequences in 10 mM phosphate buffer at pH 7.4 without (a,c,e) and with 50% TFE (b,d,f). a) and b) show **D2D**, **D2L**, and **D2R**, c) and d) show **D2D** compared with **13** and **14**, and e) and f) show **D2D** compared with **15** and **16**. All spectra were recorded at room temperature on a Jasco J-815 in 1 mm quartz cuvettes. The y-axis shows the ellipticity signal normalized to the UV absorbance for all sequences.

© 2019 by the authors. Submitted for possible open access publication under the terms and conditions of the Creative Commons Attribution (CC BY) license (http://creativecommons.org/licenses/by/4.0/).
